# Crystal structure and receptor-interacting residues of MYDGF — a protein mediating ischemic tissue repair

**DOI:** 10.1038/s41467-019-13343-7

**Published:** 2019-11-26

**Authors:** Rebecca Ebenhoch, Abbas Akhdar, Marc R. Reboll, Mortimer Korf-Klingebiel, Priyanka Gupta, Julie Armstrong, Yining Huang, Lee Frego, Irina Rybina, John Miglietta, Anton Pekcec, Kai C. Wollert, Herbert Nar

**Affiliations:** 1 Boehringer Ingelheim Pharma GmbH & Co. KG, Birkendorfer Str. 65, 88397 Biberach an der Riss, Germany; 2 Division of Molecular and Translational Cardiology, Department of Cardiology and Angiology, Hannover Medical School, Carl-Neuberg-Str. 1, 30625 Hannover, Germany; 3 Boehringer Ingelheim Pharmaceuticals, Inc., 900 Ridgebury Road, Ridgefield, CT 06877 USA

**Keywords:** X-ray crystallography, Drug discovery, Biologics, Cytokines, Signal transduction

## Abstract

Myeloid-derived growth factor (MYDGF) is a paracrine-acting protein that is produced by bone marrow-derived monocytes and macrophages to protect and repair the heart after myocardial infarction (MI). This effect can be used for the development of protein-based therapies for ischemic tissue repair, also beyond the sole application in heart tissue. Here, we report the X-ray structure of MYDGF and identify its functionally relevant receptor binding epitope. MYDGF consists of a 10-stranded β-sandwich with a folding topology showing no similarities to other cytokines or growth factors. By characterizing the epitope of a neutralizing antibody and utilizing functional assays to study the activity of surface patch-mutations, we were able to localize the receptor interaction interface to a region around two surface tyrosine residues 71 and 73 and an adjacent prominent loop structure of residues 97–101. These findings enable structure-guided protein engineering to develop modified MYDGF variants with potentially improved properties for clinical use.

## Abstract (web summary)

Myeloid-derived growth factor (MYDGF) is an angiogenic growth factor with therapeutic potential for ischemic tissue repair and the receptor is still unknown. Here the authors present the crystal structure of human MYDGF and identify its functional epitope through mutagenesis studies.

## Introduction

One of the major causes for morbidity and mortality worldwide still remains acute myocardial infarction (MI)^1^. Acute MI is mediated by a thrombotic occlusion of a coronary artery, which leads to progressive cell death in the nonperfused tissue^2^. This triggers an inflammatory response, which leads to scar formation and loss of viable tissue. Severe alteration of tissue architecture in the left ventricle can cause chamber dilatation, contractile dysfunction and heart failure^3^.

A previously functionally uncharacterized protein, named myeloid-derived growth factor (MYDGF), has shown to improve tissue repair and heart function in rodent models of MI^4^. In comparison to wild-type mice, MYDGF-deficient mice develop larger infarct scars and more severe contractile dysfunctions^4^. Treatment with recombinant MYDGF helps to protect and repair the heart after acute MI. Development of protein-based therapy would be a novel and promising therapeutic approach for cardiac repair^5^ and potentially also for ischemic repair in other tissues.

MYDGF, previously named c19orf10 (chromosome 19 open reading frame 10), is a poorly characterized protein, which was identified as a paracrine-acting protein that is secreted by monocytes and macrophages^4^. MYDGF is evolutionarily highly conserved and resides in the endoplasmic reticulum (ER), in the Golgi and extracellularly^6^. According to its primary amino acid sequence, MYDGF does not belong to any known cytokine or growth factor family. Human MYDGF has 142 amino acids and consists of an N-terminal 31-residue-long signal peptide and the MYDGF protein sequence containing a C-terminal KDEL-like ER retention sequence^6^. Even though MYDGF’s effects on cardiac tissue repair have been revealed and downstream signaling pathways have been identified^4^, the MYDGF receptor(s) or binding partners mediating these effects remain unknown. MYDGF does not belong to any known growth factor or cytokine family, shows no sequence homology to any other protein and is structurally uncharacterized. Therefore, the determination of the MYDGF structure could help to bring this exceptional protein into its biological context and to get a deeper understanding of its mode of action.

Here, we report the X-ray structure of the therapeutically promising protein MYDGF. Furthermore, MYDGF's functionally relevant receptor-binding epitope is precisely characterized by determination of the epitope of a neutralizing antibody and by utilizing functional assays to study the activity of surface patch-mutations and N- and C-terminally tagged MYDGF variants.

## Results

### MYDGF shares no sequence homology with other proteins

Local sequence alignment using BLAST searches shows that MYDGF shares no sequence similarity to any other protein^7^. MYDGF’s closest homolog (5HQA; glycoside hydrolase) in Protein Data Bank (PDB) has <30% sequence identity. Similarly, more elaborate Hidden Markov Model-based approaches for homology detection, like HHpred^8^, also failed to find proteins with high sequence coverage. Except for the N-terminal signal peptide, which targets proteins for translocation across the ER membrane into the classical secretory pathway^9^, MYDGF does not contain any known domains or motifs, according to the SMART and PROSITE algorithms^10,11^. Taken together, MYDGF appears to be a protein with uncommon structural features.

### De novo determination of MYDGF’s structure

Since no conclusions could be drawn from the analysis of the protein sequence on the mode of action of MYDGF, we hoped to obtain more detailed information about the affiliation to a protein family from protein structure determination. Recombinant full-length MYDGF, lacking the N-terminal signal peptide, was crystallized using sitting-drop vapor diffusion methods. MYDGF crystals diffracted to 1.6 Å. Due to the lack of initial start models, the structure of MYDGF was determined by Hg-SAD phasing by derivatization of a trigonal crystal form using Hg(II)I_2_. Data collection and refinement statistics are compiled in Supplementary Table 1.

**Fig. 1:**
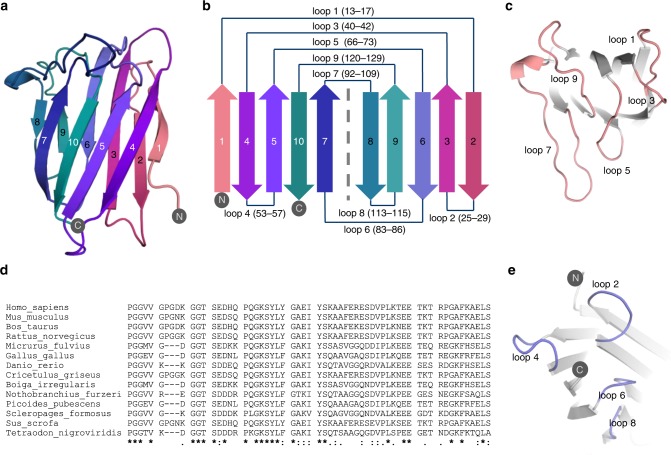
Crystal structure and sequence conservation of MYDGF. **a** Cartoon representation of the MYDGF structure with gradual coloring from red (N-terminus) to green (C-terminus). **b** Topology diagram of MYDGF displays the connectivity between the 10 β-sheets. **c** The elongated loops on the *top face* are highlighted in red. **d** Multiple sequence alignment of the loop 1–9 regions distinct MYDGF species (full alignment in Supplementary Fig. 5). High sequence conservation is observed especially in loops 1, 3 and 5. Panel **e** shows MYDGF's *bottom face* with the short loops (blue) and both termini.

The MYDGF structure consists of 10 antiparallel β-strands forming two β-sheets, which are packed face to face to each other, forming a β-sandwich (Fig. 1a). The β-strands are connected by loops of 3–18 amino acid lengths and enclose a hydrophobic interior. The structure contains several sheet-exchanged Greek key motifs, which are a common feature of many β-sandwich proteins^12^. The loop structure of MYDGF is highly asymmetric. Short, hairpin-like loops are located on one end of the β-sheets, while three elongated loops (loops 5, 7 and 9; Fig. 1c) are on the other end. We refer to the area holding the elongated loops as the *top face* (Fig. 1c) and to the area carrying the shorter loops as the *bottom face* (Fig. 1e). The surface area of the top face is therefore much larger compared to the bottom face. Loops 5 and 7 are the top face’s most prominent and elongated loops. These loops laterally protrude in a 90° angle over the β-sandwich core. Two large surface areas are spanned by both β-sheets. The β-sheet plane consisting of β-strands 1, 4, 5, 10 and 7, which is located on the side toward which the loops are projecting, is from now on called *front face*, whereas the opposite β-sheet consisting of β-strands 2, 3, 6, 9 and 8 is named the *back face*. A single disulfide bond between cysteine residues 32 and 61 of β-strands 3 and 5, respectively, bridges the opposing β-sheets. No posttranslational modifications are observed within the MYDGF structure. The N- and C-termini are located at the bottom face.

The asymmetric unit (ASU) contains two molecules of MYDGF forming a close contact. Analysis using PISA suggests that this dimer with a buried surface area of 1726 Å^2^ may be a physiologically relevant form. However, analysis of MYDGF by analytical size-exclusion chromatography clearly indicates that MYDGF is monomeric in solution (Supplementary Fig. 1). Furthermore, we were able to crystallize MYDGF in a distinct, orthorhombic crystal form, in which MYDGF is clearly monomeric and does not exhibit the above dimer arrangement (Supplementary Fig. 2). We conclude that in contrast to other β-sandwich proteins, MYDGF is a monomeric cytokine.

### MYDGF cannot be assigned to any known protein fold class

The MYDGF structure has similarities to other β-sandwich proteins such as members of the immunoglobulin and jelly roll fold or βγ-crystallin superfamily, but the connectivity of the single strands and the number of strands differ from all known protein classes. Figure 1b shows the topology diagram of MYDGF model. Fold recognition, structural alignment and the classification tools GESAMT and CATH failed to identify proteins possessing the same fold. A small, non-functional subdomain of a 500-kDa family 98 glycoside hydrolase (TIGR4) from *Streptococcus pneumoniae* (PDB accession code 2WMF) exhibits the same connectivity of β-strands and belongs to the chondroitinase Ac topology^13^. However, the subdomain differs from MYDGF in β-strand length, total number of β-strands, and a superposition of corresponding C_α_ atoms results in an RMSD value of 4.1 Å (Supplementary Fig. 3).

We conclude that MYDGF adopts a previously unknown β-sandwich topology. All identified proteins showing structural similarity to MYDGF are functionally very heterogeneous. Therefore, no connection between folding and function can be established. Likewise, due to the lack of close structural similarity to other cytokines or receptor ligands, no conclusions can be drawn about potential MYDGF receptors.

### Loop 5 is the most highly conserved region on MYDGF

Database searches using BLAST have identified MYDGF/C19orf10 homologs in 18 vertebrate species, including amphibians and fishes (PMID: 17362502). Overall MYDGF shows a high sequence conservation across all vertebrate species. The most conserved regions in vertebrates are located in loops 3 and 5 (Fig. 1d). A BLAST search across all species identified a total of 164 MYDGF homologs. The alignment shows that loop 5 is the only sequence stretch showing up to 90% consensus (Supplementary Figs. 4 and 5).

### Functional studies enable analysis of receptor epitope

We employed a human coronary artery endothelial cell (HCAEC) migration assay to assess the biological activity of recombinant wild-type and mutant MYDGF proteins (PMID: 28931551). In this assay, MYDGF shows improvement in wound healing and cell migration comparable to treatment using vascular endothelial growth factor A (VEGFA)^4^. We further employed a complementary approach for orthogonal validation of MYDGF activity. In this assay, MYDGF induces endothelial cell migration in a p38-dependent manner. Effects can be thus reversed in a dose-dependent manner upon treatment with a p38 selective kinase inhibitor (SB203580) (see below). We used this functional readout to identify the relevant surface epitope of MYDGF that constitutes the putative receptor binding site and is key to its biological function.

First, we explored whether the location of purification tags at the N- and C-termini has an influence on MYDGF function, possibly by sterically blocking receptor interaction^14^. Human tag-free MYDGF and N- or C-terminally His-tagged mouse MYDGF were tested. We found that MYDGF variants with or without tags (Supplementary Fig. 6) had comparable biological activities. Therefore, it is likely that the interaction area between MYDGF and the receptor is not located at MYDGF’s bottom face from which both termini protrude.

### Identification of a MYDGF neutralizing antibody

**Fig. 2:**
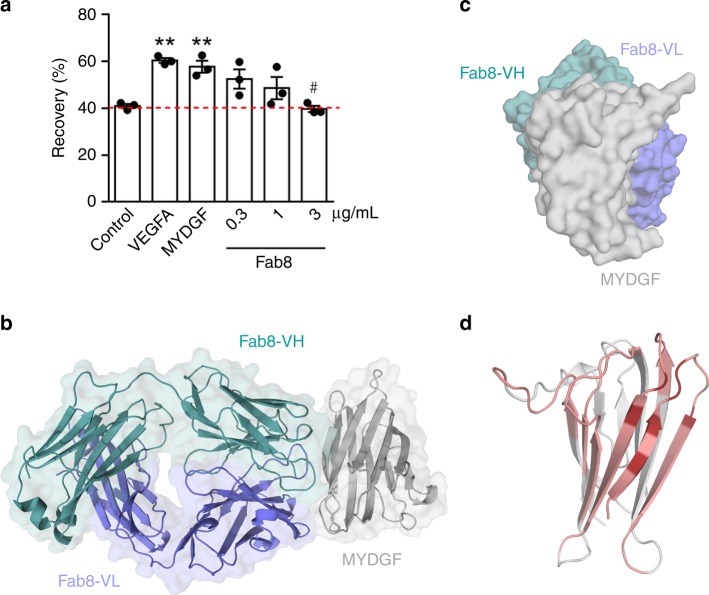
Endothelial cell migration assay and crystal structure of MYDGF-Fab8 complex. **a** Migration assay quantifying HCAEC monolayer recovery 16 h after scratch injury in the absence (control) or presence of vascular endothelial growth factor A (VEGFA, 50 ng/ml) or wild-type MYDGF (100 ng/ml) in the absence or presence of control IgG (10 µg/ml), or Fab8 (at indicated concentrations). Fab8 shows neutralizing effects. **P < 0.01 vs. control; ^#^*P* < 0.05 vs. MYDGF + IgG; *n* = 3; one-way ANOVA + Dunnett's; SEM error bars. Supplementary Fig. 11 shows the endothelial cell migration assay using Ab8. **b** Cartoon and surface representation of MYDGF (gray) and neutralizing Fab8 variable light (blue) and variable heavy chain (turquois). **c** Asymmetrical binding of Fab8 to MYDGF sterically blocks a large area on MYDGF top and front face for binding of the unknown receptor (same color coding as above). **d** The MYDGF-Fab8 epitope is colored in dark red. All residues, which are possibly shielded by the Fab8 and therefore not accessible for receptor recognition, are colored in salmon.

Next, we screened multiple antibodies targeting MYDGF for their ability to modulate the effects of MYDGF on cell migration. Antibody 8 (Ab8) and Ab8’s Fab fragment (Fab8) reversed MYDGF’s effects in a dose-dependent manner and therefore acts as a neutralizing antibody (Fig. 2a). This classification of the mode of action enables for using them as tools to characterize the receptor–interface. Structural methods like X-ray crystallography and hydrogen deuterium exchange (HDX) are able to determine the binding epitope of antibodies or their Fab-fragments. Together with the information about the mode of action, surface residues on the antigen can then be excluded or nominated as potential residues involved in receptor recognition.

### Fab8’s recognition of MYDGF shields large surface areas

We determined the crystal structure of Fab8 bound to MYDGF at 1.6 Å resolution. The recognition site of Fab8 on MYDGF is located at the edge of the β-sandwich, which is formed by the N-terminal β-strand 1 (Fig. 2b). This is consistent with HDX data showing lower exchange rates in the stretch of amino acid residues 1–34 (Supplementary Figs. 7 and 8).

The epitope of Fab8 includes residues from β-strands 1 (Ala8, Asp10, Arg12, Pro13), 2 (His18) and 4 (Asn43 and Gln45); the paratope interactions mainly involve residues from the variable heavy chain (Tyr33, Phe101, Thr103, Tyr106, Asp102, Asn 57, Asn55, Glu54), while the variable light chain (Phe91, Gly93) is only marginally involved in binding and protrudes over MYDGF’s top and front sides. The interaction area between MYDGF and Fab8 extends over about 680 Å^2^, the heavy chain contributing more than 70% to this interaction area. Supplementary Fig. 9 shows residues involved in the epitope and paratope interface. Since binding of Fab8 most probably blocks receptor binding and thereby neutralizes MYDGF’s biological activity, the asymmetrically localized epitope suggests that the surface regions of MYDGF that are positioned opposite to the epitope might not be relevant receptor recognition sites. These areas include larger parts of the back face and the β-sandwich edge formed by β-strands 7 and 8 (Fig. 2c). Conversely, both the asymmetric fashion of Fab8 binding and the relatively small size of MYDGF compared to Fab8, identify larger portions of the MYDGF surface as potentially relevant for receptor binding (Fig. 2d). These include large areas on the top and front faces of MYDGF that may be blocked by Fab8. For cytokine–receptor interactions, large surface-area interactions are typical: for example, both the epidermal growth factor receptor extracellular domain and the insulin receptor ectodomain bind their cognate ligands, EGF and insulin, in domain interfaces^15,16^. Here, the modulating proteins are highly shielded by the receptors and a large surface area on the ligands is inaccessible. In conclusion, based on the co-structure of MYDGF with the neutralizing Fab8, we cannot precisely define the receptor binding epitope of MYDGF, but we can roughly localize those areas that are more likely to be involved in receptor recognition. The top, side and front faces of MYDGF are inaccessible for receptor binding due to peripheral or direct steric hindrance of Fab8. These three faces on MYDGF could therefore be involved in receptor recognition.

### Mutagenesis of surface patches lead to inactive MYDGF

For a precise determination of MYDGF residues that are interacting with the unknown receptor, we designed 20 MYDGF variants each carrying a set of surface residue mutations. Instead of the commonly used alanine-scanning method, we decided to mutate not only single amino acid residues to alanine. Instead, entire surface patches of neighboring amino acids, all 2–4 residues in number and covering the entire MYDGF surface, were mutated. Furthermore, we decided to change individual residues not necessarily to alanine only, but to more drastically altered amino acids, e.g. with opposite charges or distinct steric demands, since interacting receptor ligand surface areas need to be complementary in shape and charge. This method has the advantage that fewer mutants are required to scan the entire protein surface and the potential effects on biological activity are expected to be much greater, thus facilitating unambiguous detection of altered protein activity in functional assays. Supplementary Table 2 shows the mutated residues of all variants.

**Fig. 3:**
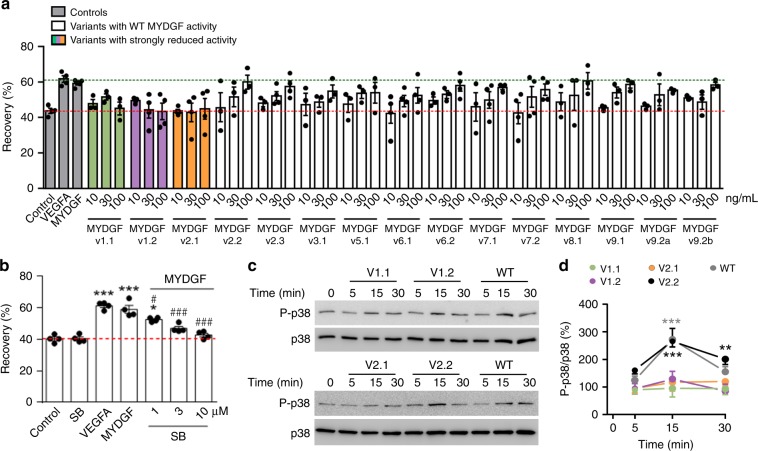
MYDGF patch mutations and effects of wild-type MYDGF and variants on p38 activation in HCAECs. **a** Migration assay quantifying HCAEC monolayer recovery 16 h after scratch injury in the absence (control) or presence of vascular endothelial growth factor A (VEGFA, 50 ng/ml), wild-type MYDGF (100 ng/ml) or MYDGF mutants (at indicated concentrations). Biologically active MYDGF mutants are colored black, while the inactive mutants are colored green, purple and orange (SEM error bars). **b** MYDGF (100 ng/ml) induced endothelial cell migration is mediated by p38-MAPK. This effect can be reversed in a dose-dependent manner upon treatment with a p38 selective kinase inhibitor (SB203580) (10 µM). VEGFA (50 ng/ml) was used as a positive control (***P < 0.001, *P < 0.05 vs. Control; ^###^*P* < 0.001, ^#^*P* < 0.05 vs. MYDGF; SEM error bars). **c** Western blot analysis of MYDGF wild-type and variant stimulated HCAECs. **d** Densitometric analysis of Western blot in **c**. P-p38 signals were referenced to total p38 signals and summarized. Wild-type MYDGF and v2.2 show similar P-p38/p38 ratios, while v1.1, v1.2 and v2.1 show a reduction of p38 phosphorylation (***P < 0.001, **P < 0.01 vs. Control; SEM error bars).

The designed MYDGF variants show a high coverage and good distribution of mutated residues on the MYDGF surface (see below). Three out of 20 MYDGF variants, namely v1.1, v1.2 and v.2.1, displayed strongly reduced activity in the endothelial cell migration assay, while the remaining variants behaved similarly to wild-type MYDGF (Fig. 3a). Five variants were excluded from functional testing due to insolubility issues or reduced thermal stability. To verify these results, the activity of the three inactive MYDGF variants were tested in the p38-activation assay to measure the downstream signaling. Wild-type protein MYDGF and one active MYDGF variant were used as controls. The active variant v2.2 triggered increased levels of phosphorylated p38, comparable to wild-type MYDGF, while use of all inactive MYDGF variants did not lead to an increase in p38 phosphorylation (Fig. 3c, d), confirming the cell migration assay data.

We used nanoDSF to measure protein thermal stability and exclude that protein misfolding was responsible for the three variants’ biological inactivity. All three variants had comparable or even higher melting points than wild-type MYDGF (Supplementary Fig. 10), thus implying proper folding.

### Identification of receptor epitope on MYDGF’s top face

**Fig. 4:**
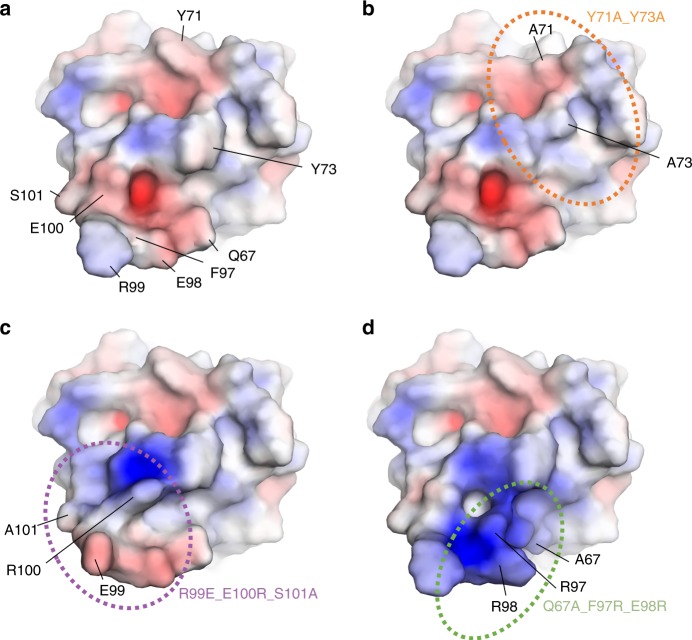
Visualization of the effect of the MYDGF inactive variants on surface conformation and electrostatics. Electrostatic potential was calculated in PYMOL. The individual panels show the distribution of charges on the surface (negative charges—red; positive charges—blue) of **a** wild-type MYDGF, **b** Y71A_Y73 variant, **c** R99E_E100R_S101A and **d** Q67A_F97R_E98R.

**Fig. 5:**
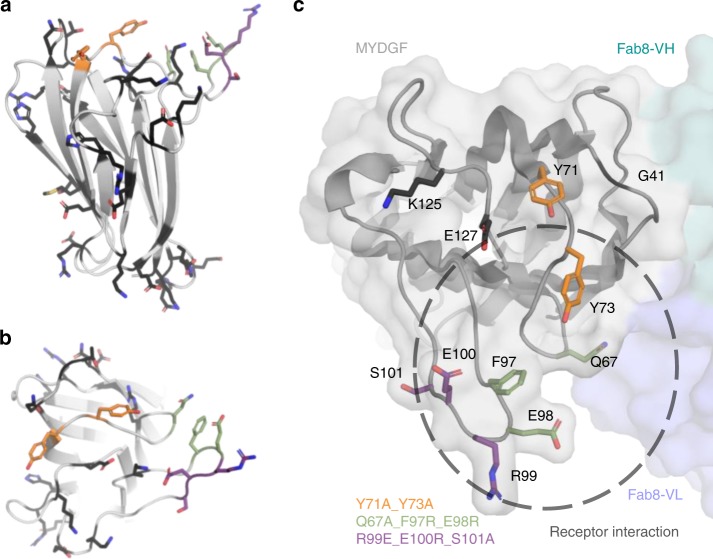
MYDGF patch mutations and MYDGF receptor epitope. **a** Distribution of all (black) and the functionally important mutated residues (green, purple and orange) on the MYDGF surface in side view. **b** Distribution of all (black) and the functionally important mutated residues (green, purple and orange) on the MYDGF surface in top view. **c** Variants Q67A_F97R_E98R (green), R99E_E100R_S101A (purple) and Y71A_Y73 (orange) are located on the top face of MYDGF on loops 5 and 7. Fab8 (blue and turquois) is recognizing the β-sandwich edge of MYDGF including the N-terminal residues 1–18. A circle with dashed line marks the area on MYDGF, which facilitates interaction to the unknown MYDGF receptor.

Notably, the three surface patches that are mutated in the three inactive MYDGF variants are located in close proximity to each other (Fig. 4). The patches lie on MYDGF’s top face on loops 5 and 7 (Fig. 5c), which are both protruding from the β-sandwich core domain above MYDGF’s front face. MYDGF patch mutants Q67A_F97R_E98R (green in Fig. 5) and R99E_E100R_S101A (purple in Fig. 5) belong to the class of variants which were designed to exhibit a highly repulsive effect toward possible receptor binding due to the inversion of charges. Figure 4a shows the calculated electrostatic potential of the wild-type MYDGF surface in comparison to the inactive MYDGF variants. Charge exchange mutants are similar in size and should not primarily impact sterical requirements of receptor binding, but would abolish binding if they were involved in salt bridge interactions with the receptor. The fact that these mutants are biologically inactive variants of MYDGF may therefore be explained either by direct involvement of the respective amino acid side chains in receptor binding or by secondary effects such as longer-range Coulomb electrostatic repulsion. The information as to which of the mutated residues are directly involved in the receptor interaction is therefore limited.

In contrast, the exchange of tyrosine to alanine in variant Y71A_Y73A (orange in Fig. 5) should have none of these limitations, since it imposes lower steric demands and is expected to be neutral with regard to changes in surface electrostatics compared to wild-type MYDGF (Fig.  4a, b).

### Tyr71/73 could be the key residues for receptor interaction

The finding that this variant is inactive leads us to the assumption that residues Tyr 71 and Tyr 73 directly interact with the receptor and most probably are key residues with a decisive influence on the receptor binding affinity. Both residues are highly conserved in MYDGF ortholog sequences (Fig. 1d, Supplementary Figs. 4 and 5). Tyr71 is identical across all species, while Tyr73 is highly conserved, and replaced by the aromatic amino acid phenylalanine in non-mammalian sequences. Tyrosine side chains are often involved in protein–protein interactions. The high proportion of aromatic residues, in particular tyrosine and tryptophan, involved in protein–protein interactions has been noted before and reflects a general property of antibody epitopes^17–19^. Interestingly, the mutations G41W and K125S_E127R (Fig. 5c), which are located on loops 3 and 9 and therefore adjacent to Tyr 71 and Tyr 73, have no effect on MYDGF’s activity (mutants v.2.2 and v9.2a in Fig. 3a).

This observation suggests that the receptor interaction is more likely to take place in the front/protruding part of the top face and indicates that Tyr73 may be the key residue for receptor interaction.

## Discussion

Our work has revealed the structure of MYDGF, a recently identified angiogenic growth factor showing therapeutic potential for ischemic tissue repair^4^. The enormous potential of MYDGF, proven for ischemic tissue repair after acute MI, could also be applicable to other tissues. There is a large variety of diseases, like e.g. ischemic stroke or inflammatory joint disease, which cause serious tissue damage through insufficient perfusion and thrombotic occlusions^20,21^. The potential of MYDGF to positively affect other ischemic diseases needs to be evaluated and would help to obtain a more complete understanding of the breadth of it’s therapeutic applications.

MYDGF is an exceptional growth factor in multiple aspects. Besides the fact that MYDGF shares no sequence similarity to any other protein, we have shown here that MYDGF does not display structural homologies to any other protein and cannot be unambiguously assigned to any protein fold class. Therefore, we were not able to derive any conclusion relating to its affiliation to a protein family or any hints toward the identification of a potential MYDGF receptor from its protein structure.

MYDGF patch variants helped to identify the functional regions on the MYDGF surface that are responsible for interaction with the putative receptor. Mutagenesis studies carry the risk that changes in single amino acids on the surface of a protein can lead to rearrangements of the immediate environment. For the set of mutants we introduced, we cannot fully exclude any unwanted change in protein dynamics or conformation. However, based on biophysical evidence for preserved thermal stability of the inactive variants compared to wild-type, we presume that the overall 3D structure should be largely conserved. Further, for the introduction of point mutants in loop regions involving charge reversal residue swaps, it is plausible to assume that the orientation of the side chains projecting toward the solvent should be retained, resulting in minimal structural and dynamic alterations. Finally, we refrained from introducing amino acids, like glycine and proline, which have the potential to alter backbone torsion angles, flexibility and overall conformation. An intriguing outcome of our mutagenesis work was that of the 15 patch mutants studied, 12 had similar functional activity as the wild-type protein. We therefore suggest that the abolished activity of the three patch mutants is exclusively due to their inability to bind to the receptor.

We identified a clearly defined area involving MYDGF’s top face, in particular loops 5 and 7, to form the receptor binding interface. This conclusion is fully consistent with the analysis of the structure of the MYDGF-Fab8 complex, which restricts the receptor recognition surface area to the top, side and front faces of MYDGF. While the epitope of Fab8 does not overlap with the proposed receptor epitope on the top face, the proximity of the neutralizing Fab8 epitope to this site suggests competitive inhibition via peripheral steric clashes, which hinder the approach of MYDGF binding proteins (Fig. 5c). Furthermore. we postulate a potential receptor binding hot spot around residues Tyr71 and Tyr73 (Fig. 5c). In contrast to the remaining inactive MYDGF variants, neither changes in the electrostatic potential nor increase in steric demands were introduced in the Y71A_Y73A variant. The introduction of alanine instead of tyrosine leads to reduction of the steric claim on the epitope leaving a void area. If the receptor paratope offered pockets for the tyrosine side chains to bind to, they would be left empty by the variant, which should drastically lower ligand affinity. We conclude that while the inactivity of the two variants Q67A_F97R_E98R and R99E_E100R_S101A strongly suggest participation of the corresponding residues in the receptor epitope, lack of activity of the Y71A_Y73A variant points to the Tyr71/73 region as being the central hub for receptor binding.

Our elucidation of MYDGF’s structure and putative receptor-interacting substructure will enable structure-guided protein engineering to improve its drug-like properties, including stability, potency, availability and solubility of MYDGF. For instance, as MYDGF is a relatively small 16-kDa protein that may be prone to an enhanced rate of renal clearance in comparison to more common therapeutic proteins like IgGs. This is related to the permeability threshold of the kidney glomerular membrane, which retains proteins larger than albumin in the plasma^22^. Size adaption of MYDGF is therefore a logical strategy for half-life extension. In addition, the design of fusion proteins, or use of scaffolding proteins for loop crafting, or rigidification of flexible loop regions are established techniques in protein engineering to overcome limitations in drug delivery^23–26^. Additionally, improved pharmacodynamics may be achieved by increasing the affinity between ligand and receptor. This can be done by random, large-scale mutagenesis, which requires a tremendous screening effort. In this context, the information generated within this work can be advantageous in this context, since both knowledge of MYDGF’s 3D structure and, in particular, the identification of its functionally important residues, potentially involved in receptor binding, will greatly reduce the number of relevant residues that need to be probed.

Identification of the potential MYDGF receptor(s) or MYDGF-binding partners would be extremely important to gain further knowledge on the mode of action of MYDGF. The neutralizing and non-neutralizing antibodies as well as the inactive MYDGF variants described here will serve as powerful controls in pull-down assays for receptor identification, which are typically delivering a high number of false-positive hits. Structural information on the receptor–ligand complex would not only shed light onto the complex biology of MYDGF, but also at the same time give information relevant to rational, structure-based design to improve MYDGF’s therapeutic properties.

In summary, this work has revealed the previously unknown 3D structure of MYDGF, which cannot be assigned to any known protein fold class. In addition, we provided insights into the MYDGF receptor-binding epitope. This gained knowledge, together with the developed tools for receptor identification studies, should stimulate further efforts to identify the MYDGF receptor and help to elucidate the role of MYDGF beyond repair processes for MI, as well as its biological and pathophysiological implications in other tissues. Together, this knowledge should advance the development of MYDGF-based therapies for ischemic tissue repair to fully exploit the enormous therapeutic potential of MYDGF.

## Methods

### Expression and purification of recombinant MYDGF

The cDNA of human MYDGF was codon optimized for mammalian expression (Supplementary Table 3). A 6xHis Tag followed by a tobacco etch virus (TEV) cleavage sequence was introduced between the signal peptide and the N-terminus of the mature secreted MYDGF protein. The wild-type MYDGF amino acid sequence is provided in Supplementary Table 3. The entire ORF was gene synthesized with XbaI and BamHI restriction sites, which was then used for restriction-enzyme-based cloning into pTT5 vector. The secreted protein was captured from the conditioned supernatant of MYDGF expression vector transfected human embryonic kidney cell line, HEK 293-6E (RRID:CVCL_HF20), cells using Ni-NTA resin (Novagen) and Econo-columns (Bio-Rad). Fractions eluted with imidazole (250 mM in PBS; pH 7.4) containing the recombinant protein (as analyzed by SDS-PAGE) were pooled. TEV protease (1.2 × 105 units; Promega) and β-mercaptoethanol (final concentration, 5 mM) were added, and the reaction mixture (40 ml) was dialyzed overnight at room temperature against 8 l of PBS with 5 mM β-mercaptoethanol (pH 7.4). The TEV cleavage protein mixture was then subjected to negative Ni-NTA column purification. Eluate fractions containing the unbound target protein (as shown by SDS-PAGE) were collected and concentrated using Vivaspin 20 centrifugal concentrators (5000 MWCO; Merck). The protein was further purified by size exclusion chromatography (SEC) with an ÄKTAFPLC system (GE Healthcare) using a 5 × 90 cm Superdex 200 column (GE Healthcare) equilibrated in PBS (pH 7.4) and a flow rate of 1 ml/min PBS (pH 7.4) to remove TEV protease and other impurities. Fractions containing cleaved MYDGF were identified by UV absorbance and pooled. The concentration of the purified protein was determined by UV absorbance applying the specific extinction coefficient of the cleaved protein (absorbance at 280 nm (1 mg/ml) = 1.21).

The DNA constructs for MYDGF variants were designed like wild-type MYDGF, having an N-terminal signal peptide, followed by a 6xHis Tag, a TEV cleavage site and the mature MYDGF sequence with the distinct point mutations. MYDGF variants were expressed and purified exactly like WT MYDGF, besides the N-terminal 6xHis Tag was not cleaved.

### Analytical size exclusion chromatography

The purified MYDGF was assessed for monomer quality by SEC. Ten micrograms of protein was injected into an analytical SEC column, 200 Å, 1.7 µm, 4.6 mm × 150 mm (GE Healthcare), using a Waters UPLC-system. The system was run at 0.5 ml/min for 5 min and the mobile phase consisted of 50 mM NaPO_4_, 200 mM l-Arginine, pH 6.8. The elution of MYDGF was detected via absorbance measurements at 280 nm using a UV absorbance detector. BioRad molecular weight standards were used to calculate the molecular weight based on retention times.

### Expression and purification of recombinant AB/Fab8

Tool antibody 8 was made by Cell Essentials Inc., Boston, MA, using standard hybridoma methology. The mouse hydridoma cells for antibody 8 were used to recover variable gene sequences using the 5′ RACE kit and custom in-house primers targeting the VH and VL regions of mouse antibodies (Supplementary Table 3). The variable genes VH and VL of the Ab8 were used to make chimeric fragment antibody (Fab) by gene synthesis and cloning the genes into pTT5 vectors containing constant regions for human CH1 and human kappa, respectively. 6X His Tag was included at the end of C-terminus for CH1 to enable purification of the Fab.

The Fab was produced by transient transfection of a human embryonic kidney suspension cell line, HEK 293F (Thermo Fisher, R79007), using PEI transfection reagent. Linear PEI MAX (Mw 40,000) (Polysciences: cat. # 24765-2) was used at 1:3 ratio of DNA:PEI with a total of 0.9 µg/ml DNA (HC:LC; 1:1). The supernatant containing secreted Fab was harvested 6 days post transfection and filtered before purification. The supernatant was supplemented with 10 mM Imidazole and pH was adjusted to 7.5 prior to loading onto the Ni-NTA Affinity column (XK 16/20 packed with 10 ml Ni NTA Agarose matrix). Loading was performed at 4–8 °C; 2 ml/min flow using Äkta Pure followed by a wash of 10 column volumes with Buffer A (1× PBS, pH 7.4) and 4% Buffer B (1× PBS, 0.5 M imidazole, pH 7.4). The Fab was eluted off the column with 4–60% Buffer B in linear gradient elution over 10 column volumes. The elution was collected as 4 ml fractions and analyzed by SDS-PAGE prior to pooling the fractions which contained the protein of interest. The Fab was further purified by loading the Ni NTA purified fractions onto a SEC column (Hi load 26/600, Superdex 200 pg column) equilibrated with the formulation buffer, 1× PBS, pH 7.4, at a flow rate of 1 ml/min. The fractions were collected and analyzed by SDS-PAGE prior to pooling.

### MYDGF structure determination

For crystallization, the protein buffer was exchanged by SEC (Superdex™ 200 increase 10/300 GL, GE Healthcare) run using 40 mM Bicine (pH 7.5), 150 mM NaCl and 20 mM KCl and concentrated up to 50 mg/ml using Amicon centrifugal filters (3 kDa cutoff). All crystals were obtained by sitting drop vapor diffusion using 96-well 3-drop SWISSCI plates (MolecularDimensions). The protein was mixed in a 1:1 ratio with reservoir solution and was equilibrated against the reservoir. Crystals were obtained from a reservoir solution containing 48% M1K3350 (12% w/v PEG 1000, 12% w/v PEG 3350, and 12% v/v MPD; original solution Molecular Dimensions), 0.09 M NPS (30 mM sodium nitrate, 30 mM sodium phosphate dibasic and 30 mM ammonium sulfate; original solution Molecular Dimensions), 0.1 M MMT buffer (pH 6.4) (0.02 M dl-malic acid, 0.04 M MES monohydrate, 0.04 M Tris). Crystals grew at 4 °C after 5 days. All crystals were flash-frozen in liquid nitrogen.

For heavy atom derivatization, crystals were soaked for 6 h at 4 °C in reservoir solution supplemented with 10 mM Hg(II)I_2_ and then rapidly washed in a drop reservoir solution to get rid of Hg(II)I_2_ before flash-freezing into liquid nitrogen.

X-ray diffraction data were collected at the Swiss Light Source (SLS; Villigen, Switzerland) at the PXIII and PXI beamline, and processed with the autoPROC pipeline^27^ using the XDS package^28^ resolution cutoffs were calculated using STARANISO^29^. Data processing statistics are listed in Supplementary Table 1. The structure of MYDGF was determined from Hg(II)I_2_-derivatized crystals. Single anomalous dispersion (SAD) data were recorded at the Hg peak wavelength of 0.99 Å. Five datasets with different phi and chi angles were collected from a single crystal, resulting in reasonable anomalous signal up to 2.2 Å. Identification of the heavy atom substructure, phasing and density modification was performed using autoSharp^30^ and SHELXD^31^. The model of MYDGF was manually built using Coot^32^ and the resulting model was improved by iterative rounds of manual rebuilding and refinement with autoBuster^33^. The final refinement was performed against a dataset that originated from a crystal that was not derivatized and diffracted up to 1.6 Å. The crystals belonged to space group P3_1_21 (Hg_MYDGF, native MYDGF) and contained two monomers per ASU. The final native MYDGF model and structure factors have been deposited in the PDB (accession code 6SVK).

### MYDGF-Fab complex structure determination

Fab8-MYDGF complex was formed by mixing MYDGF with excess of Fab8 and purifying the complex by SEC (Superdex 200 Increase 10/300 GL, GE Healthcare) with crystallization buffer (40 mM bicine (pH 7.5), 150 mM NaCl, 20 mM KCl). The Fab8-MYDGF complex was subsequently concentrated up to 35 mg/ml using Amicon centrifugal filters (30 kDa cutoff) and crystallization trials were set up as described above. Crystals were obtained using 0.1 M trisodium citrate (pH 5.5) and 20% PEG 3000 and cryoprotected by addition of 30% glycerol to the mother liquor. The crystal diffracted up to 1.6 Å resolution, belonged to the space group P1 and contained six Fab8-MYDGF complex molecules per ASU. Data- and refinement statistics are listed in Supplementary Table 1. The phases were obtained by molecular replacement (Phaser-MR^34^) using the MYDGF structure and the variable region of a mouse IgG Fab fragment (1IQW) as search models. Further processing, refinement and model building was done as described above. The final model and structure factors have been deposited in the PDB (accession code 6SVL).

### Hydrogen deuterium exchange

For HDX (hydrogen/deuterium exchange mass spectrometry) experiments, MYDGF was analyzed alone (control, 4 µl MYDGF with concentration of 0.39 mg/ml) and with antibody present at approximately an equimolar ratio (mixed sample, 3.79 µl of antibody with concentration of 3.7 mg/ml mixed with 0.21 µl of MYDGF with concentration of 7.5 mg/ml). All sample handling was performed by a LEAP HDX PAL system. To identify peptides, the control sample was incubated with H_2_O buffer (H_2_O 10 mM sodium phosphate pH 7.4). For exchange experiments, the control and mixed samples were incubated with D_2_O buffer (D_2_O 10 mM sodium phosphate pH 7.4) at 10, 100 and 1000 s time points (four replicates each) by the following procedure: (1) 4 µl of sample was added to 40 µl of H_2_O/D_2_O buffer. (2) The mixture was incubated at 20 °C for various time points (10, 100 and 1000 s). (3) 40 µl of the incubated sample was transferred to 40 µl of 4 °C quench buffer (4 M Urea, 0.4 M TCEP-HCl). (4) 60 µl of the quenched sample was injected onto an immobilized protease XIII/pepsin column (1:1 2.1 × 30 mm, NovaBioAssays) by flowing 200 µl/min of Mobile Phase A (99% H_2_O, 1% acetonitrile and 0.1% formic acid) for 2 min. The digested peptides were desalted on a Vanguard Pre-column (ACQUITY UPLC BEH C18, 130 Å, 1.7 µm, 2.1 mm × 5 mm, Waters) for 3 min, and then separated by liquid chromatography with an Acquity UPLC BEH C18 Column 1.7 µm, 1 mm × 50 mm (Waters) at 4 °C at a flow rate of 160 μl/min. The LC gradient solvent system was composed of mobile phase A (composition above) and mobile phase B (0.1% formic acid, 5% H_2_O and 95% acetonitrile). The percentage of mobile phase B was held at 5% for 5 min; increased from 5 to 15% at 5.6 min, to 40% at 10.4 min, to 90% at 11 min; held at 90% to 11.5 min; decreased to 5% at 12.4 min, and then held at 5% to 14 min. After chromatographic separation, the peptides were detected by the Thermo Scientific Orbitrap Fusion mass spectrometer operated in positive electrospray ionization mode. The employed method to identify the non-deuterated control peptides is a Data Dependent Acquisition (DDA) method with collision-induced dissociation (CID) and electron transfer dissociation (ETD) fragmentation. The precursor ions were detected by the orbitrap utilizing a resolution of 120,000, a minimum signal of 10,000 and an isolation width of 1.0. The S-lens RF level was set at 60%. For deuterated samples, no MS/MS data were collected. Data were then analyzed by Byonic software (Protein Metrics) to identify peptides. Uptake plots for the individual peptides are shown in Supplementary Data 1. For the searching parameters, the precursor mass tolerance was set as 10 ppm; fragment mass tolerance was set as 15 ppm for both CID and ETD; and automatic score cut off was applied. Since the purity and identity of the control sample has been confirmed by analytical size exclusion chromatography (aSEC) and intact mass analysis, the amino acid sequence of human MYDGF was used as the searching database; and the decoy database is the reversed sequence of human MYDGF. A total of 79 unique peptides (100% sequence coverage) were identified for the control sample. HDExaminer software (Sierra Analytics) was utilized to calculate deuterium incorporation. After automated analysis in HDExaminer, manual corrections were applied for replicates with large deviation in HDX values, and chromatographic peaks which have abnormal shift of retention times. The statistical analysis performed by HDExaminer employed a one-way analysis of variance^35^. The cut off/significance line was drawn on the residual plot is 0.6945 × 3 time points = 2.084 at 99% confidence level.

### NanoDSF

Wild-type MYDGF and patch-mutated MYDGF variants were diluted up to 0.1 mg/ml in PBS. NT.Plex nanoDSF Grade Standard capillaries (NanoTemper) were filled with 10-µl protein samples. Melting curves were determined in triplicates using Prometheus NT.48 by monitoring the intrinsic protein fluorescence signal as a measure of its folding state during a temperature ramp (1 °C/min increase) from 20 to 95 °C. Exemplary melting curves are shown in Supplementary Fig. 10. The melting temperature was determined by averaging the melting temperature of the triplicate measurements.

### Endothelial cell culture and functional assays

HCAECs were grown in Nunc T75 flasks (Thermo Fisher Scientific) in endothelial cell growth medium (EGM, Lonza, #CC-3162) supplemented with 10% FCS. For functional assays, cells were switched to MCDB 131 medium (Thermo Fisher Scientific, #10372019) containing 2% FCS. Branching morphogenesis was assessed on growth factor-reduced Matrigel (Corning) in 48-well plates (2.5 × 10^4^ cells/well). After stimulation, cells were stained with the membrane-permeable fluorescent dye BCECF-AM (Sigma-Aldrich) for 2 h. Digital images were acquired with an Axio Observer.Z1 fluorescence microscope (Zeiss) and analyzed using AxioVision software (Zeiss). Branching points were defined as intersections of at least three tubes and closed tubes as circular structures surrounded by tubes. Cell migration after scratch injury was analyzed in endothelial cell monolayers grown in 24-well plates. Monolayers were scratched with a 200 μl pipet tip, washed and stimulated for 16 h. Before and after stimulation, digital phase-contrast images were captured with the Axio Observer.Z1 microscope and analyzed using AxioVision software. Recovery (%) was calculated as [(cell free area at 0 h – cell free area at 16 h)/cell free area at 0 h] × 100. For signaling analysis, HCAECs were stimulated with recombinant proteins for 0, 5, 15 and 30 min, lysated in RIPA buffer (50 mM Tris, 150 mM NaCl, 1 mM EDTA, 1 mM EGTA, 50 mM NaF, 1% Triton X, 0.5% IGEPAL containing protease and phosphatase inhibitors (Roche, #4693132001, #4906845001)) and subjected to immunoblot analysis. Immunoblots were densitometrically analyzed using ImageJ v1.48.

### Reagents

Recombinant human VEGFA was purchased from R&D Systems and SB203580 from Cell Signaling Technology. Other chemicals were purchased from Sigma-Aldrich.

### Antibodies

For immunoblotting, antibodies were purchased from Cell Signaling Technology: p38 mitogen-activated protein kinase (MAPK) (p38 MAPK; clone D13E1, CST, #8690; dilution 1:1000); P-p38 MAPK (P-p38 MAPK (T180/Y182); clone 12F8, CST, #4631; dilution 1:1000).

### Statistical analyses

Data are presented as mean ± SEM. Numbers refer to the number of independent experiments. For comparisons among groups, one-way ANOVA was used. The Tukey post-hoc test was used to adjust for multiple comparisons. The Dunnett post-hoc test was used for multiple comparisons with a single control group. A two-tailed value of *P* < 0.05 indicated statistical significance. All analyses were performed with GraphPad Prism software (version 6.01).

### Reporting summary

Further information on research design is available in the Nature Research Reporting Summary linked to this article.

## Supplementary information

Supplementary Information

Description of Additional Supplementary Files

Supplementary Data 1

Reporting Summary

## Data Availability

Coordinates and structure factors for the MYDGF and MYDGF-Fab8 structures are deposited in the Protein Data Bank under accession codes 6SVK and 6SVL, respectively. The source data underlying Figs. 2a, 3a–d and Supplementary Figs. 6 and 11 are provided in a separate Source Data file. Other data that support the study are available from the corresponding author upon reasonable request.

## Source data

Source Data
