# Supplementary material for: Crystal structure and receptor-interacting residues of MYDGF — a protein mediating ischemic tissue repair
Source: Nat Commun. 2019 Nov 26;10:5379. doi: 10.1038/s41467-019-13343-7 (PMC6879528; doi:10.1038/s41467-019-13343-7)
Supplement: Supplementary file 1 — Supplementary Information [file 41467_2019_13343_MOESM1_ESM.pdf]

<u>Supplementary Information</u>

Crystal structure and receptor-interacting residues of MYDGF - a protein mediating ischemic tissue repair

R. Ebenhoch et al.

Supplementary Table 1: Data collection, phasing and refinement statistics for MYDGF structures.

	MYDGF Hg-derivatized	MYDGF native	MYDGF-Fab8
**Data collection**			
Space group	$P3_121$	$P3_121$	P1
Cell dimensions			
a, *b*, *c* (Å)	48.6 48.6 117.7	48.7 48.7 177.8	89.5 107.4 109.4
α,β,γ (°)	90.0 90.0 120.0	90.0 90.0 120.0	71.5 86.3 73.4
Wavelength	0.99	0.99	0.99
Resolution (Å)	59.2-1.6 (1.692-1.606)	59.3-1.6 (1.707-1.604)	97.8-1.6 (1.774-1.584)
R_{merge}	0.313 (5.690)	0.215 (2.299)	0.052 (0.633)
$I / \sigma I$	19.7 (1.2)	11.9 (1.2)	10.3 (1.2)
Completeness (%)	91.2 (36.7)	90.6 (40.0)	91.0 (56.2)
Redundancy	91.2 (78.6)	19.1 (15.0)	2.7 (3.0)
**Refinement**			
Resolution (Å)		42.2 - 1.6	97.8 - 1.6
No. reflections		28152	320160
R_{work} / R_{free}		22.1/25.2	18.1/20.6
No. atoms			
Protein		2130	24982
Ligand/ion		7	18
Water		180	2604
B-factors			
Protein		19.11	32.63
Ligand/ion		24.39	23.91
Water		26.07	39.10
R.m.s deviations			
Bond lengths (Å)		0.009	0.010
Bond angles (°)		1.04	1.08

*Values in parentheses are for highest-resolution shell. All structures were derived from a single crystal

Supplementary Table 2: MYDGF variants. List of the mutated residues of all MYDGF variants. The asterisk * denotes variants, which were excluded due to insolubility issues during purification or highly reduced thermal stability. Therefore, their activity was not measured.

MYDGF variant	Mutated residues	MYDGF variant	Mutated residues
v 1.1	Q67A; F97R; E98R	v 8.1	E111K; R120S; H119
v 1.2	R99E; E100R; S101A	v 9.1	K94E
v 2.1	Y71A; Y73A	v 9.2a	K125S; E127R
v 2.2	G41W	v 9.2b	K106D; E108W
v 2.3	Q39A; Q42K; N43A; R65T	v 4.1*	Y88A; Y92A
v 3.1	H18E; R12S; D10K	v 4.2*	V132W; K130S; W64A
v 5.1	E54K; H56S; Q57A	v 5.2*	D55A; H58S; K136S; K29S
v 6.1	E87K; H58S; K136E	v 8.2*	Q76A; K78Y; E80K
v 6.2	E87R; E85K; K114E	v 10.1*	T6W; H22D; P4D; E3K
v 7.1	E80R; R82E		
v 7.2	S21W; D28K; M33A		

Supplementary Table 3: MYDGF and Fab 8 sequences. List of construct sequences, and DNA sequences used for generation of MYDGF and Fab8 as well as primer sequences used for sequence recovery of variable gene of antibody 8.

Description	Amino acid sequence
Wild-type MYDGF sequence	MGWSLILLFLVAVATRVLSHHHHHHAGSENLYFQGVSEPTTVAF<br>DVRPGGVVHSFSHNVGPGDKYTCMFTYASQGGTNEQWQMSLGTS<br>EDHQHFTCTIWRPQGKSYLYFTQFKAEVRGAEIEYAMAYSKAAF<br>ERESDVPLKTEEFEVTKTAVAHRPGAFKAELSKLVIVAKASRTE<br>L
Fab 8 heavy chain sequence including signal peptide	MKCSWVIFFLMAVVTGVNSEVQLQQSGAELVRPGALVKLSCKAS<br>GFNIKDYYMHWVKQRPEQGLEWIGRIDPENSNNIYDPKFQGKAS<br>ITADTSSNTAYLQLSSLTSEDTAVYYCARGGFDTNHYAMDYWGQ<br>GTSVTVSS
Light chain sequence including signal peptide	MSVPTQVLGLLLLWLTDARCDIQMTQSPASLSVSVGETVTITCR<br>ASENIYSNLAWYQQKQGKSPQLLVYAATNLADGVPSRFSGSGSG<br>TQYSLKINSLQSEDFGNYYCQHFWGTPPWTFGGGTKLEIK

Description	DNA sequence
MYDGF sequence codon optimized for mammalian expression	gtgagcgagcccaccaccgtggcctttgacgtgaggccaggcgg<br>ggtggtgcacagcttcagccacaacgtgggtcccggcgacaagt<br>acacctgcatgttcacctacgccagccagggaggcaccaacgag<br>caatggcagatgagcctgggcacttccgaggaccaccaacactt<br>cacttgcacaatctggcgaccccagggcaagtcttacctgtact<br>tcacccagtttaaagccgaggtgagggggcagagatcgagtat<br>gcgatggcctacagcaaggccgcgtttgaaagggagtccgacgt<br>gcccctgaagaccgaggagttcgaagtgaccaagaccgccgttg<br>cgcaccggccaggtgccttcaaggcggagctttctaagctggtg<br>atcgtggccaaggccagcagaactgagctg

Description	Primers used for sequence recovery of variable gene of Ab 8
Long UPM forward primer (part of 5’ RACE kit)	5’ CTAATACGACTCACTATAGGGCAAGCAGTGGTATCAACGCA<br>GAGT 3’
Short UPM forward primer (part of 5’ RACE kit)	5’ CTAATACGACTCACTATAGGGC 3’
mouse IgG1 reverse primer	5’ TATGCAAGGCTTACAACCACA 3’
mouse kappa reverse primer	5’ CTCATTCCTGTTGAAGCTCTTGACAATGGG 3’

Supplementary Figure 1: Analytical size exclusion chromatography of MYDGF. MYDGF runs as a single symmetric, and sharp peak. >98% monomer observed with >95% recovery of protein at elution.

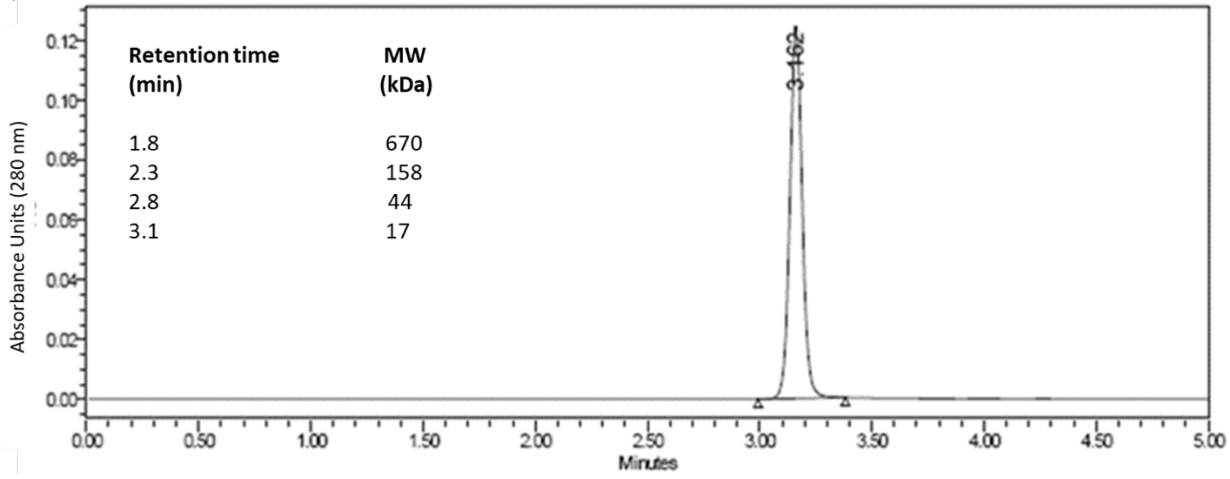

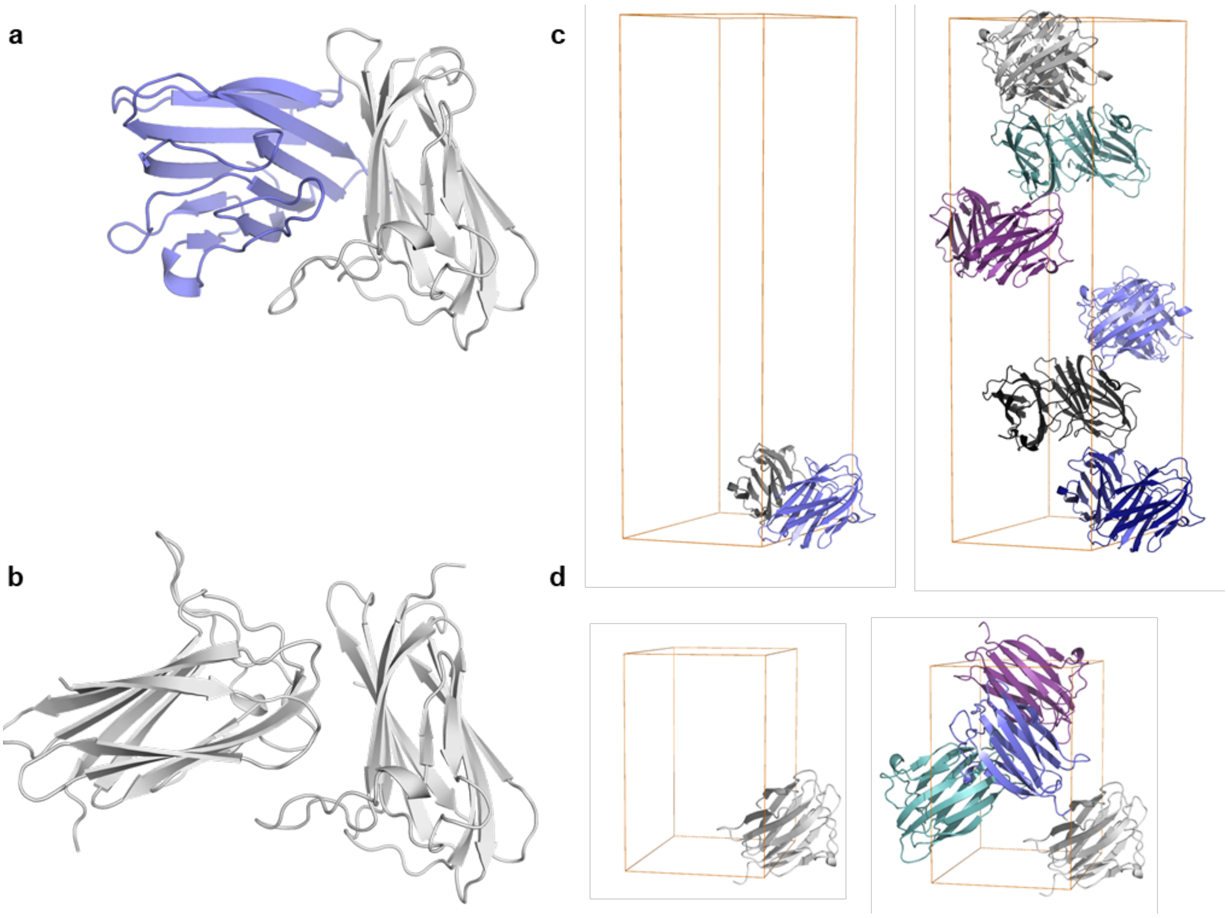

Supplementary Figure 2: Crystal packing of two distinct MYDGF crystal forms. a The trigonal crystal system has two molecules in the ASU forming close contacts. Chain A is depicted in grey and chain B in blue. The interaction of both monomers occurs mainly by linking of the extended loops. PISA analysis predicted a buried surface area of 1726 Å^2. **b** In the orthorhombic crystal form (data not shown) the ASU contains only one monomer. Chain A (gray) and its symmetry mate are packing in a different way than it was observed for the trigonal crystal. **c** The asymmetric unit of the trimeric crystal system contains two MYDGF molecules with close contacts and the unit cell (orange) of the P$3_1$21 crystal contains 12 monomers. **d** The asymmetric unit of the orthorhombic crystal system contains one MYDGF molecule and the unit cell (orange) of the P$2_12_12_1$ crystal contains 4 monomers.

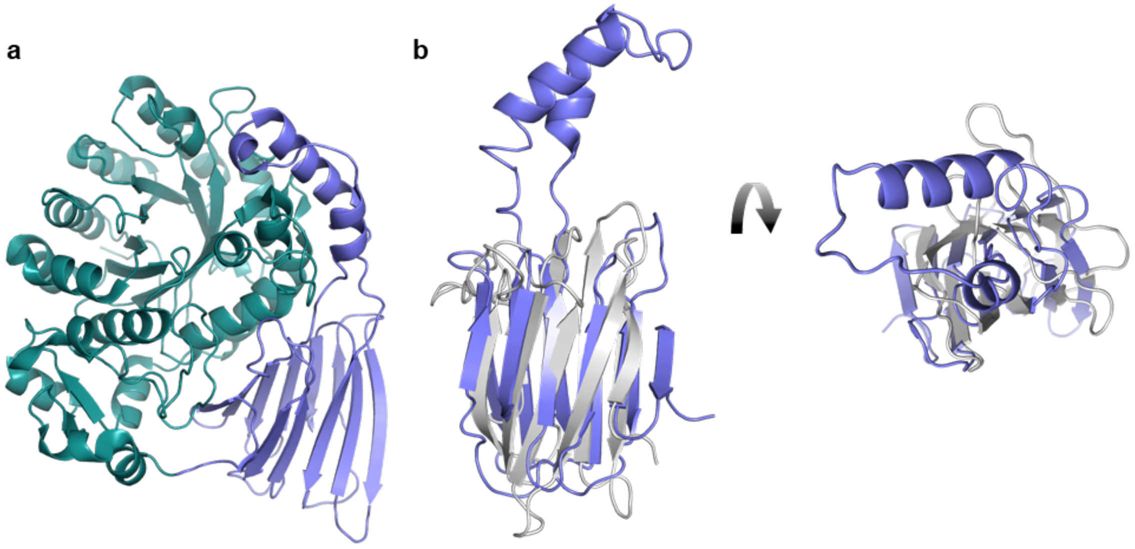

Supplementary Figure 3: Alignment of MYDGF and 2WMF. a Structure of family 98 glycoside hydrolase (TIGR4) from Streptococcus pneumoniae in cartoon representation. The β-sandwich domain is colored in blue and the remaining protein is colored in turquoise. **b** Alignment of 96 out of 166 residues of the 2WMF (blue) structure on MYDGF (grey) resulted in a Cα RMSD of 4.1 Å. 2WMF differs from MYDGF in number and length of its β-sheets and overall shape. MYDGF and 2WMF share only 3 % sequence identity.

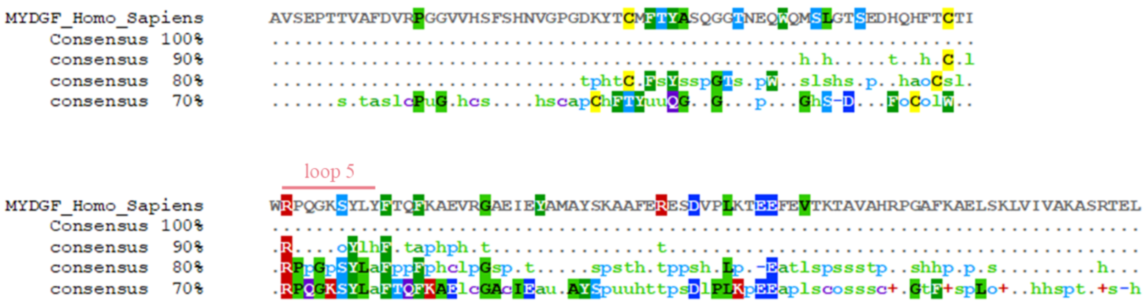

Supplementary Figure 4: Sequence alignment of MYDGF across all identified homologues. BLAST search using the human MYDGF sequence let to 164 MYDGF homologues across all species. The only region which shows 90% consensus is the region of loop 5 and surrounding residues.

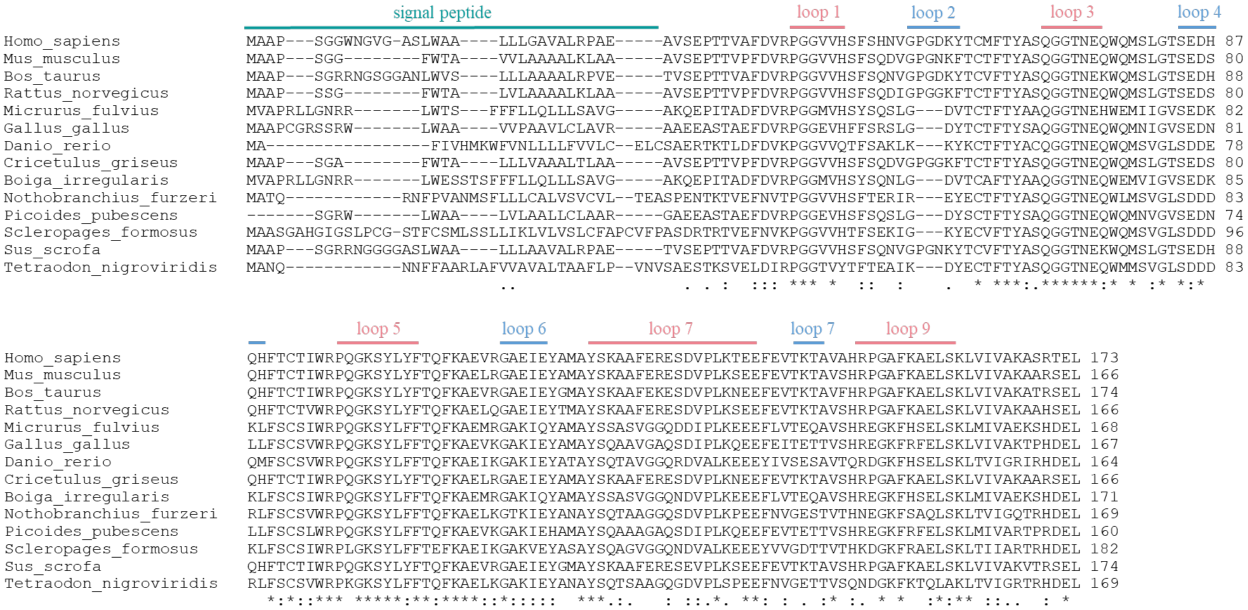

Supplementary Figure 5: Sequence alignment of MYDGF across vertebrates. MYDGF shows a high sequence conservation across vertebrate species. The most conserved loop-regions in MYDGF are loops 3 and 5, as depicted in Figure 2. The signal peptide is not as conserved as the mature protein.

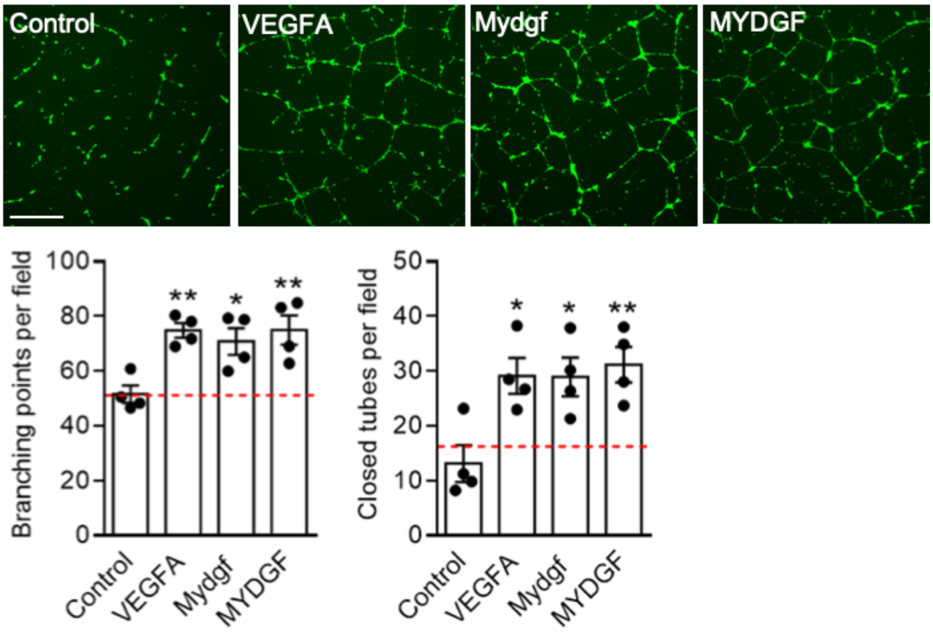

Supplementary Figure 6: Human coronary artery endothelial cell network formation. Mouse C-terminally His-tagged (Mydgf) and human non-tagged protein (MYDGF) show no difference in branching and cell networking behavior. The scale bar in the figure represents 500 µm can be applied to all microscopic images shown in this figure. The networking was evaluated after 16h stimulation with either 50 ng/ml hVEGFA, 100 ng/ml mMYDGF-His or 100 ng/ml hMYDGF. The control group was treated with diluent only (Data of N-terminally His-tagged mouse protein not shown) (**P<0.01, *P<0.05 vs. Control; SEM error bars).

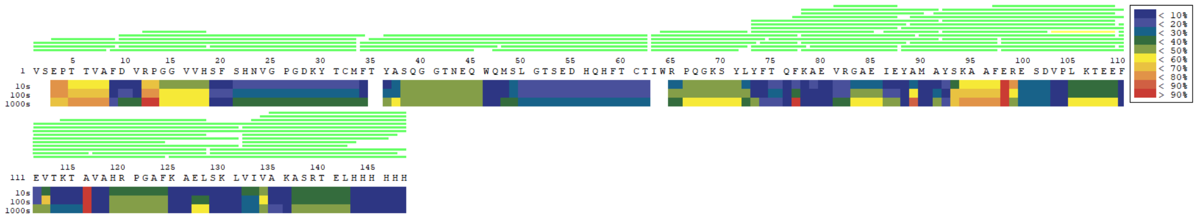

Supplementary Figure 7: Sequence coverage map of the control (MYDGF) in HDX. A total of 79 unique peptides (100% sequence coverage) were identified for the control (MYDGF) sample.

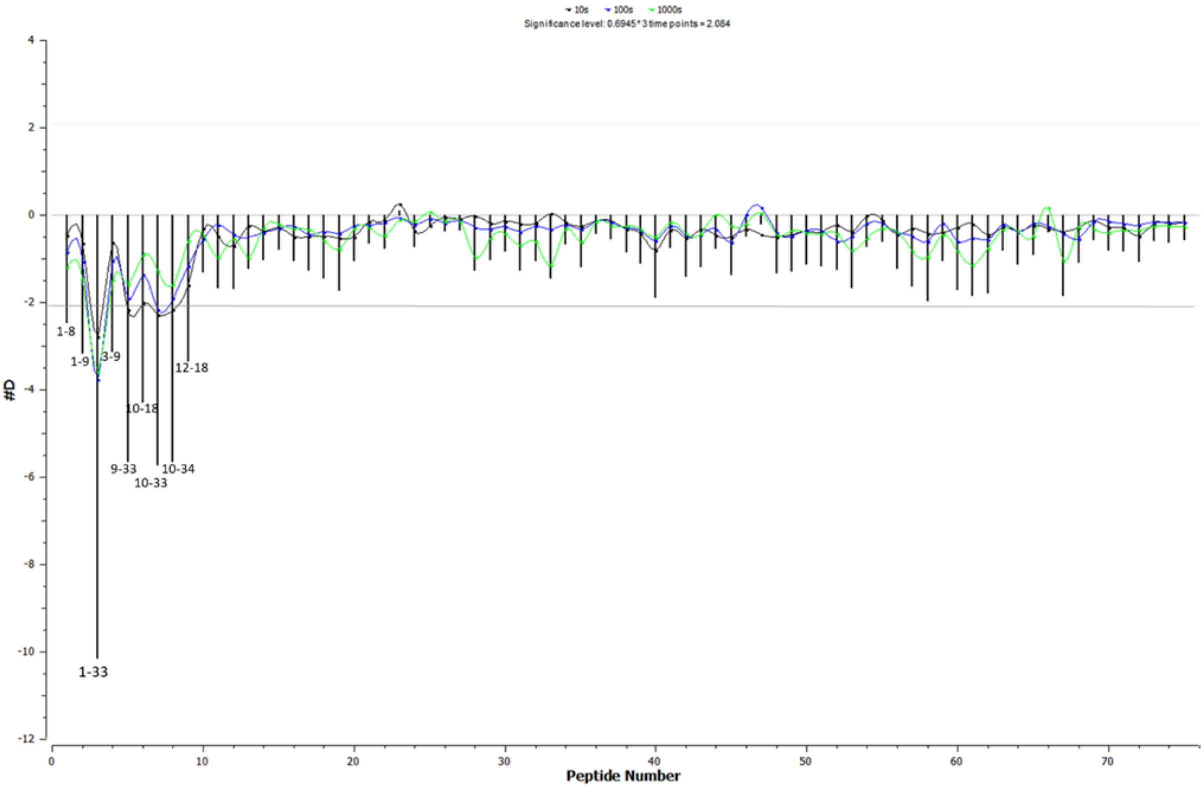

Supplementary Figure 8: Hydrogen-deuterium exchange (HDX) of MYDGF vs. the MYDGF-Ab8 complex. Differential hydrogen-deuterium exchange rates are plotted against the amino acid residue number. Amino acid residues 1-34 show the highest difference in exchange rates between unbound and Ab-bound MYDGF. Exchange rates at 10s (black), 100s (blue) and 1000s (green) are depicted. Four replicates were performed.

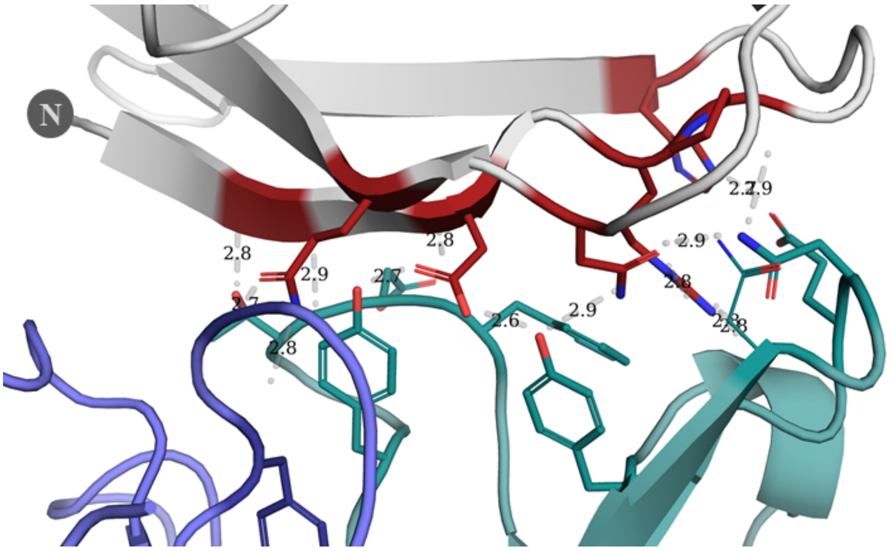

Supplementary Figure 9: Binding interface of the MYDGF-Fab8 complex. Representation of the paratope-epitope interaction between neutralizing Fab8 and MYDGF. Fab8 recognizes MYDGF via the N-terminal edge of the MYDGF β-sandwich. The variable heavy (VH) chain is colored in turquois, the variable light (VL) chain in blue and MYDGF in gray. Interacting MYDGF residues are highlighted in red.

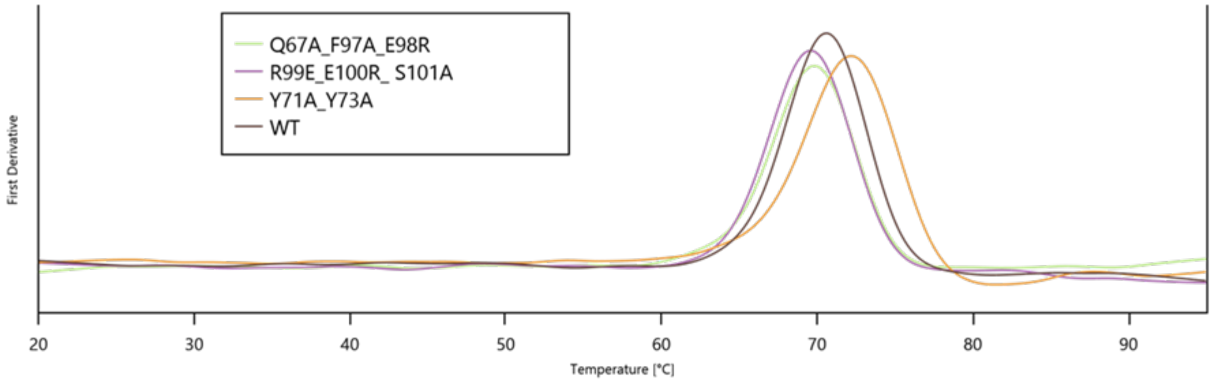

Supplementary Figure 10: nanoDSF melting curves for wild type MYDGF and its three biologically inactive variants. All proteins melt at similar and high temperatures. The melting point of wild-type MYDGF (black curve) is 70.6 °C, while the variants Q67A_ F97R_E98R (green), R99E_E100R_S101A (purple), and Y71A_Y73A (orange) melt at 69.8 °C, 69.6 °C, and 72.2 °C, respectively.

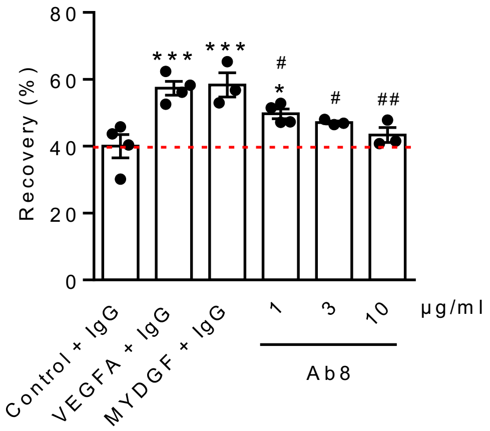

Supplementary Figure 11: Endothelial cell migration assay Using Ab8. a Migration assay quantifying HCAEC monolayer recovery 16 hours after scratch injury in the absence (control) or presence of vascular endothelial growth factor A (VEGFA, 50 ng/ml) or wild-type MYDGF (100 ng/ml) in the absence or presence of control IgG (10 µg/ml), or Ab8 (at indicated concentrations). Ab8 shows neutralizing effects. Supplementary Figure X shows the (***P <0.001, *P<0.05 vs. Control; ##P<0.01; #P<0.05 vs. MYDGF + IgG; n=3-4; one-way ANOVA + Dunnett´s; SEM error bars).
