# Supplementary material for: Crystal structure and receptor-interacting residues of MYDGF — a protein mediating ischemic tissue repair
Source: Nat Commun. 2019 Nov 26;10:5379. doi: 10.1038/s41467-019-13343-7 (PMC6879528; doi:10.1038/s41467-019-13343-7)
Supplement: Supplementary file 2 — Description of Additional Supplementary Files [file 41467_2019_13343_MOESM2_ESM.pdf]

Description of Additional Supplementary Files

File name: Supplementary Data 1

Description: HDX uptake plots for Fab8 bound and unbound MYDGF peptides. Each plot represents the average relative deuterium uptake of each peptide at different time points, determined by averaging the deuterium level found in each separate replicate experiment. Some peptides differ in deuterium incorporation between the Fab8 bound and unbound MYDGF, and some that showed no differences.
