# Supplementary figures and images for: Crystal structure and receptor-interacting residues of MYDGF — a protein mediating ischemic tissue repair
Source: Nat Commun. 2019 Nov 26;10:5379. doi: 10.1038/s41467-019-13343-7 (PMC6879528; doi:10.1038/s41467-019-13343-7)

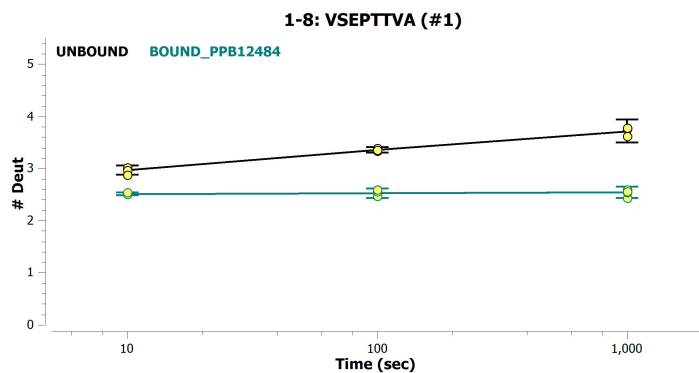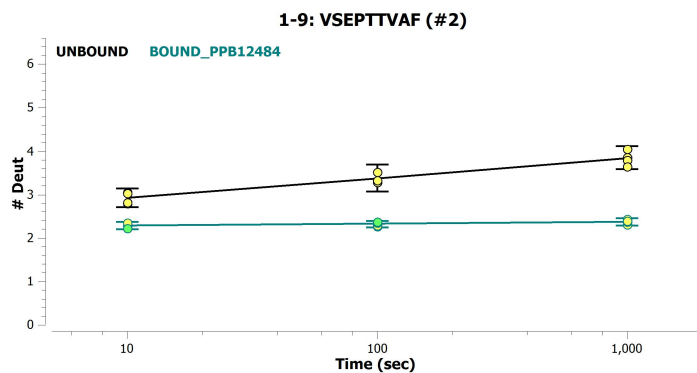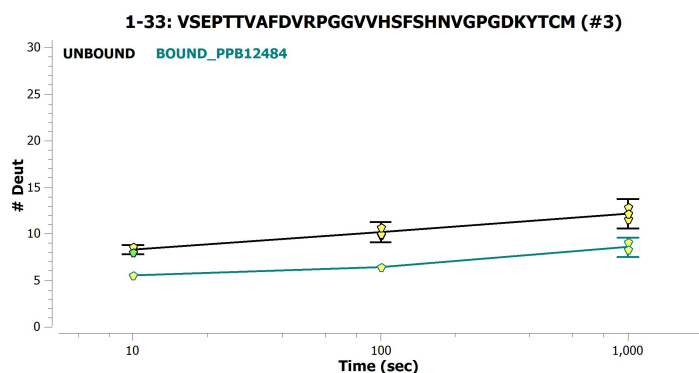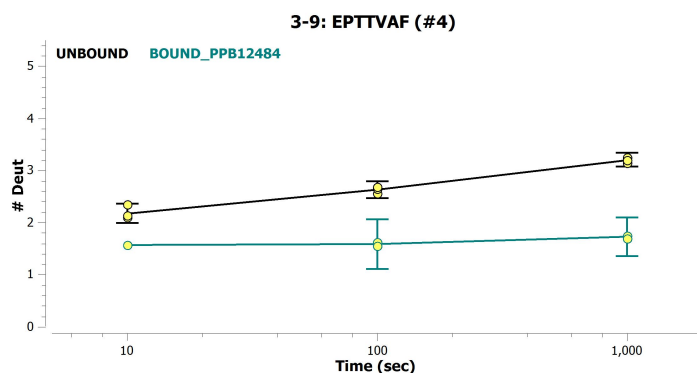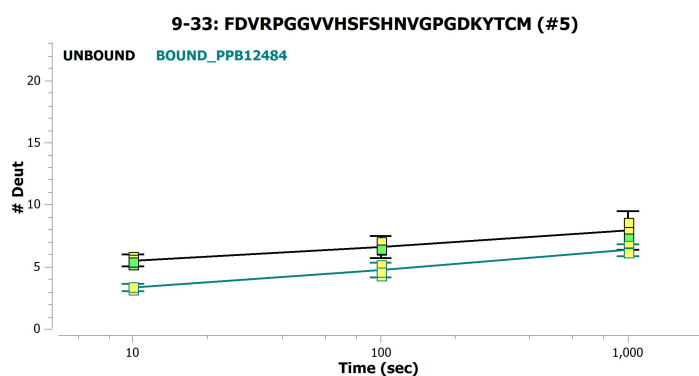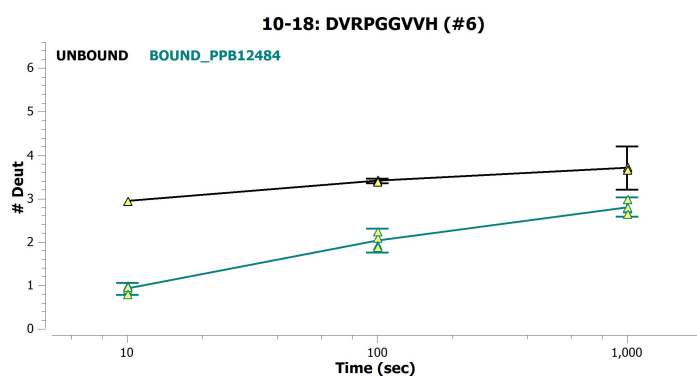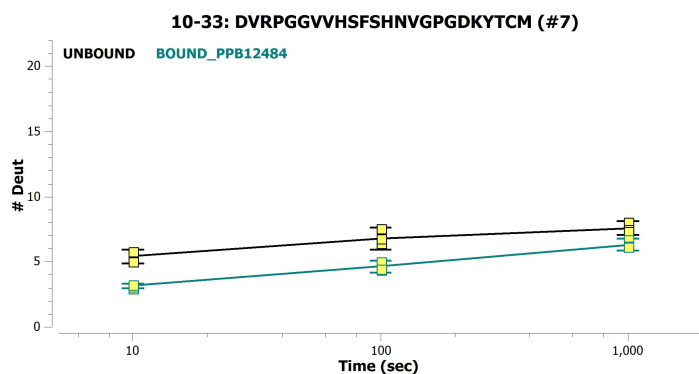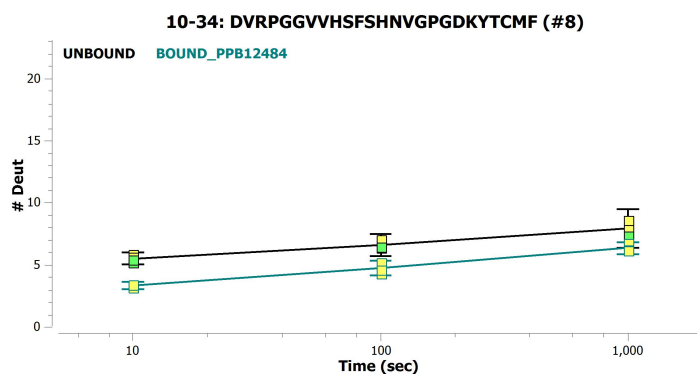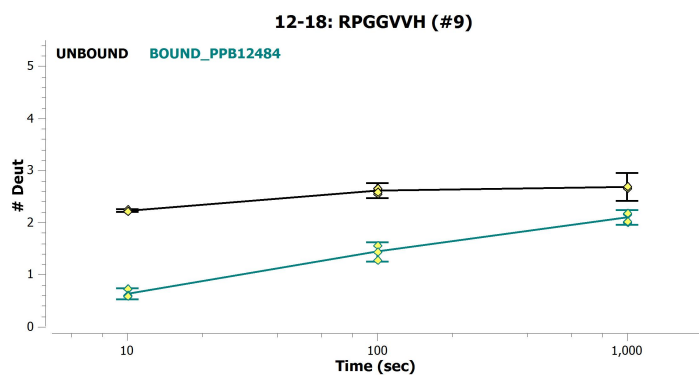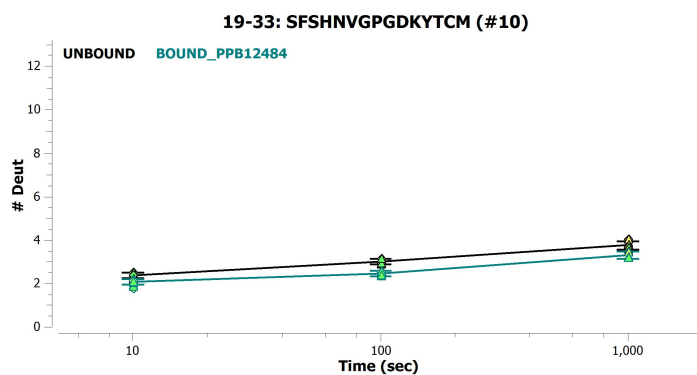

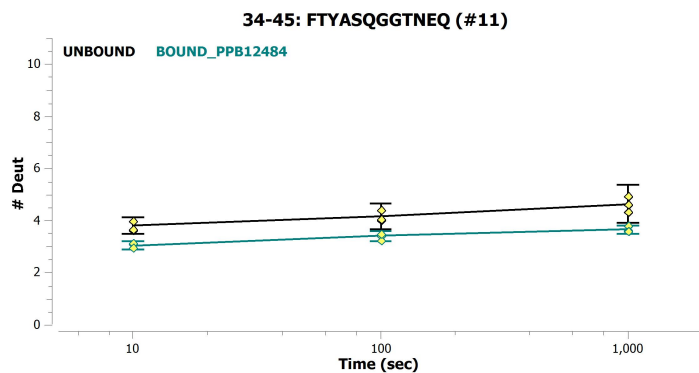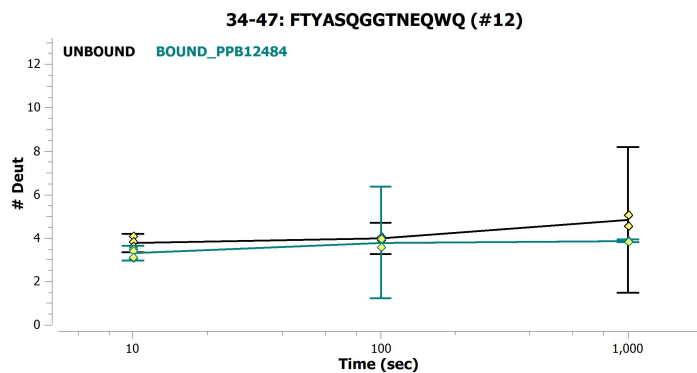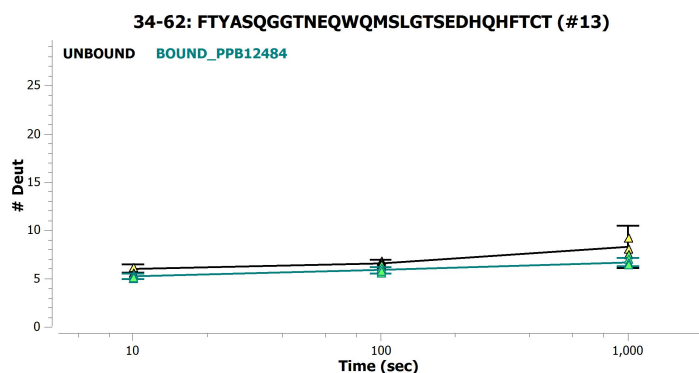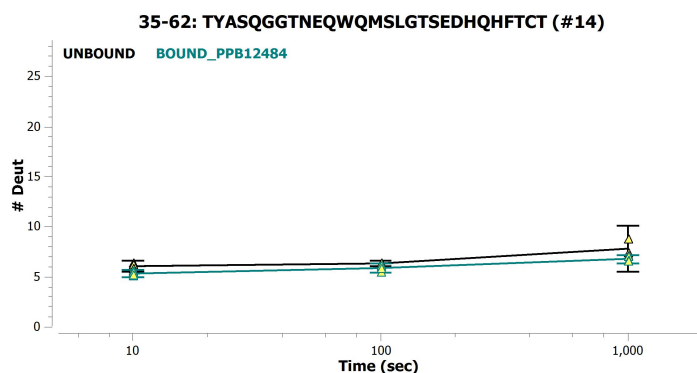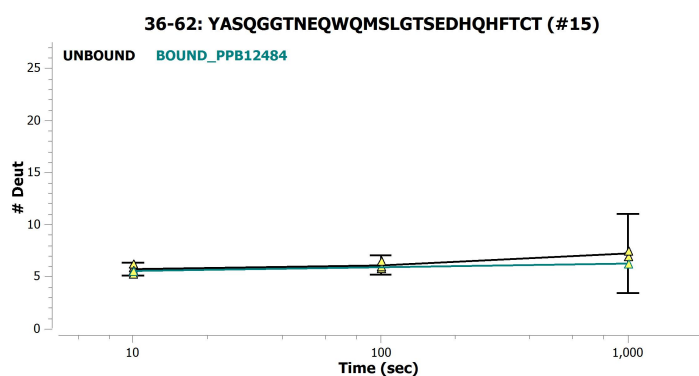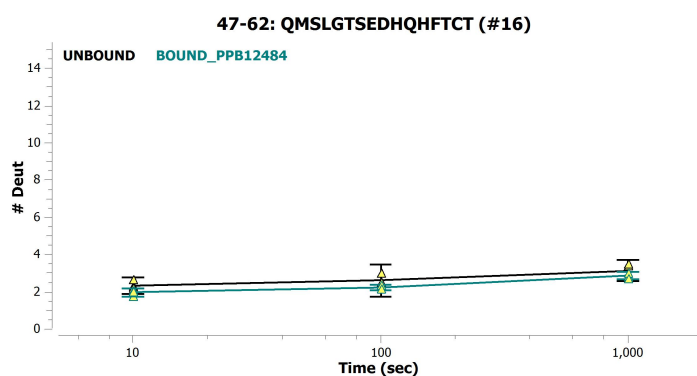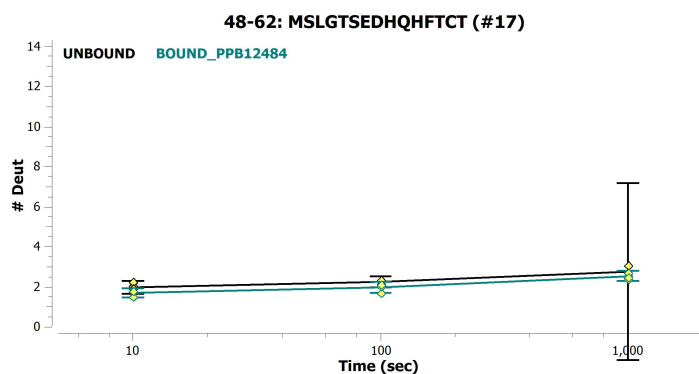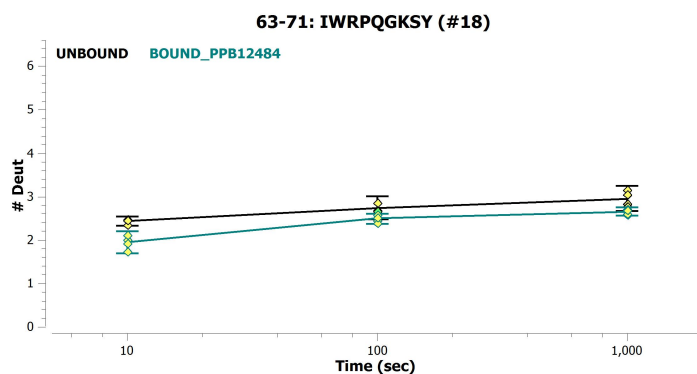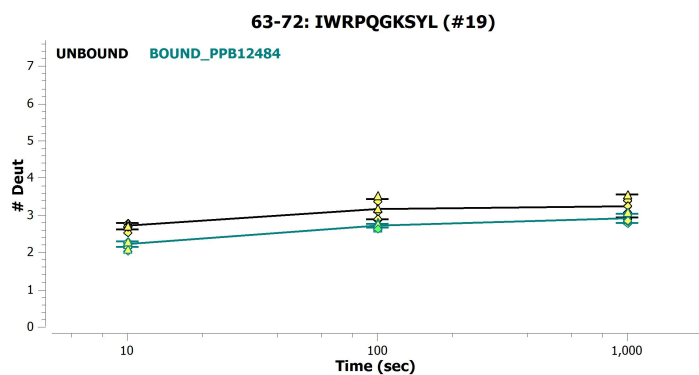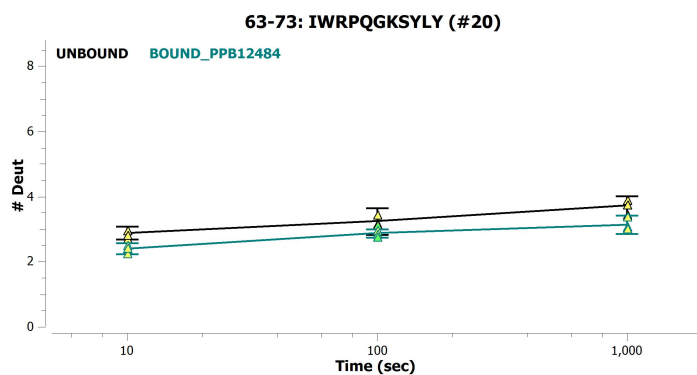

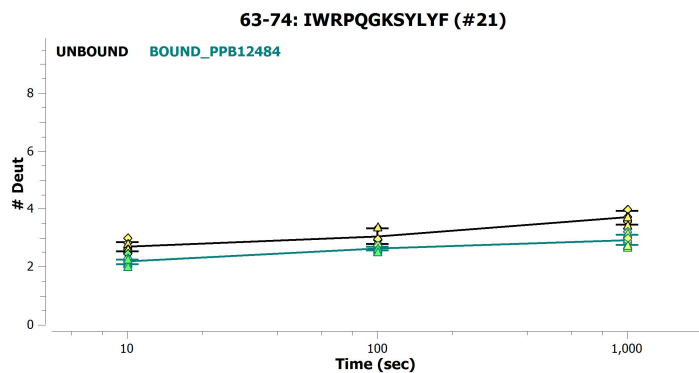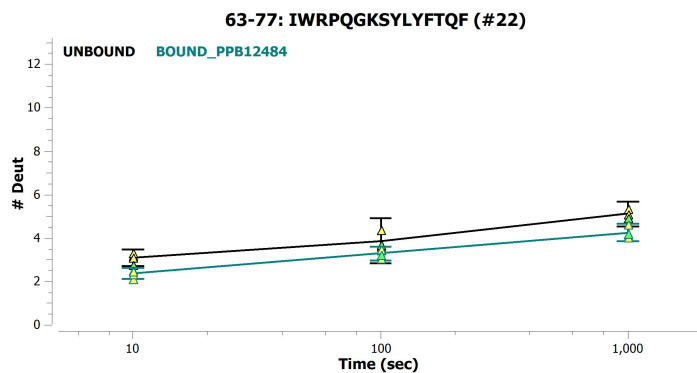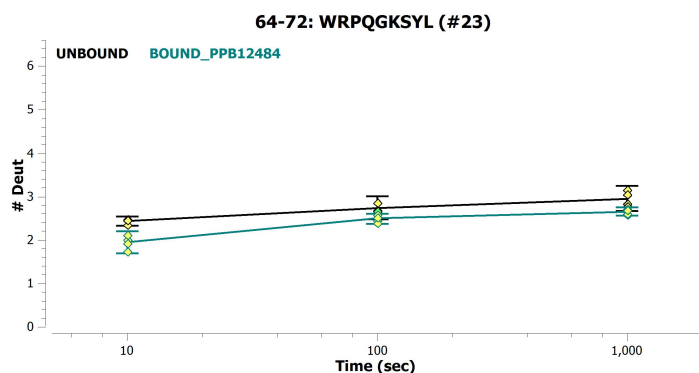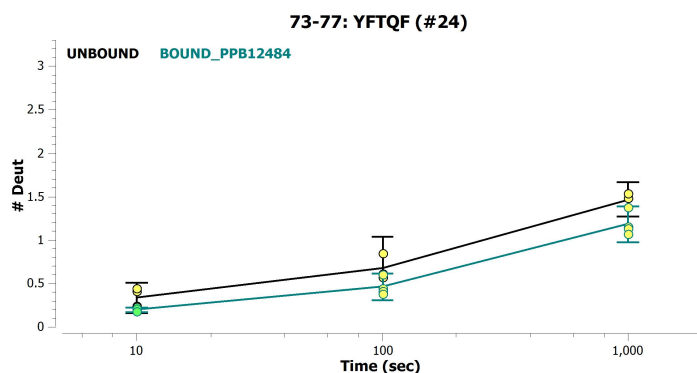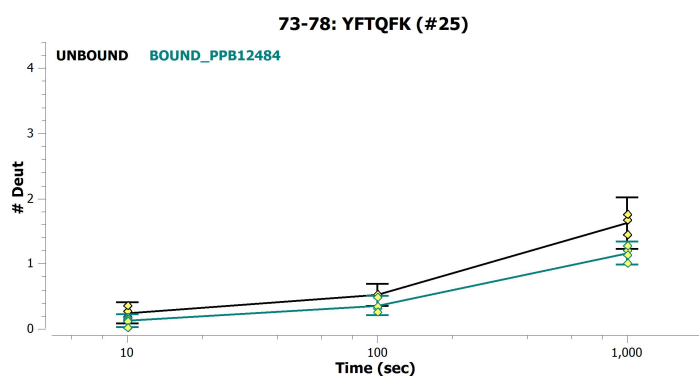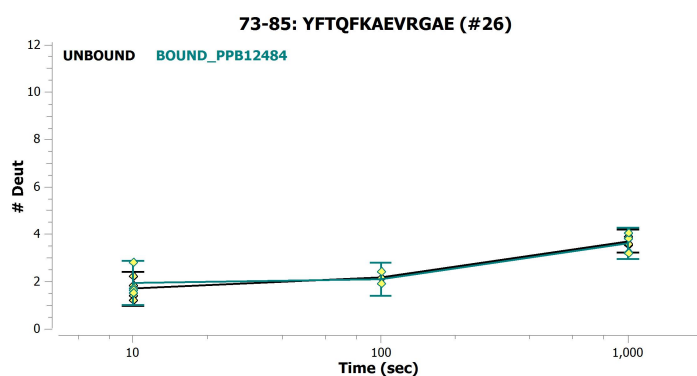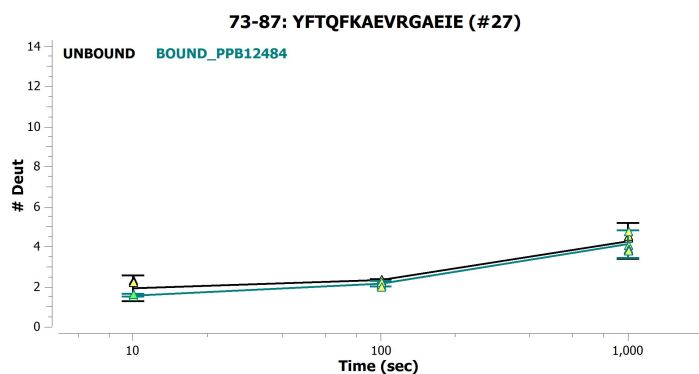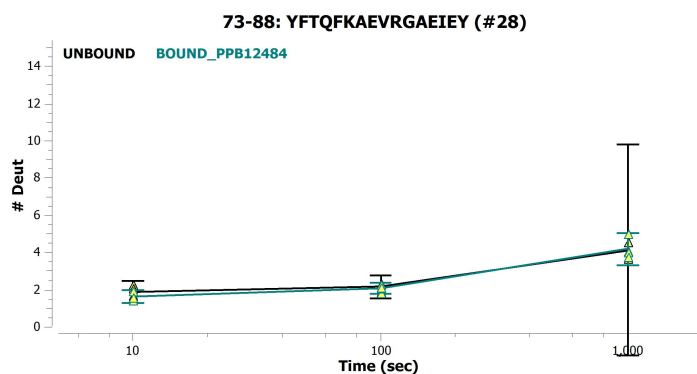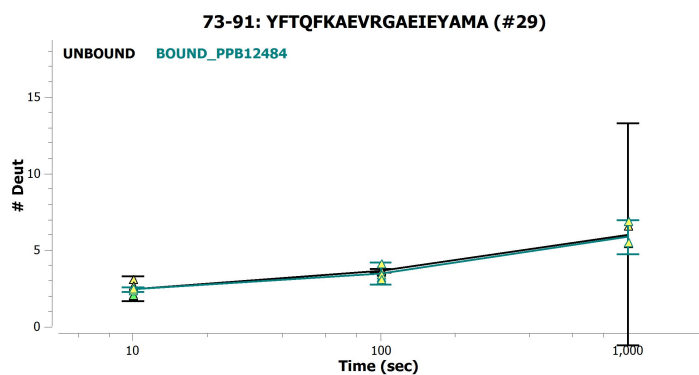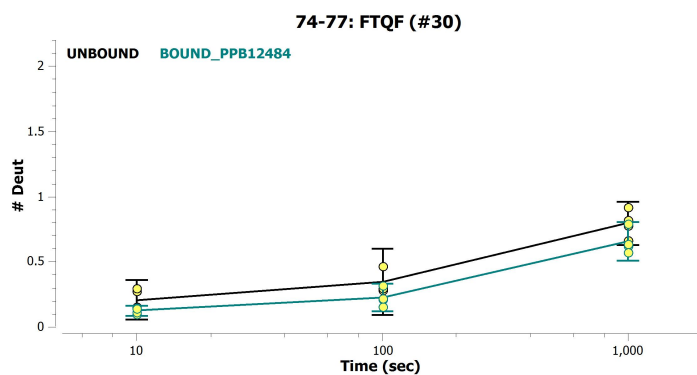

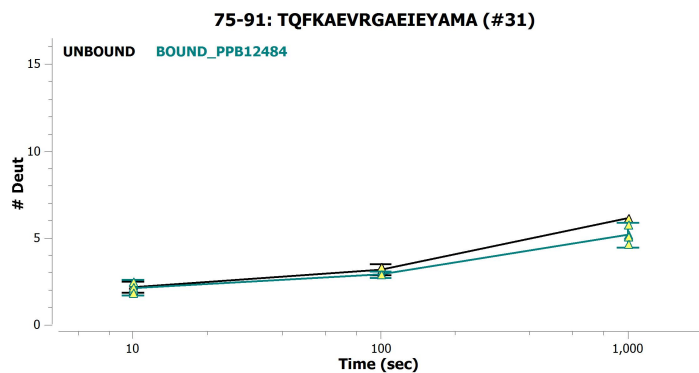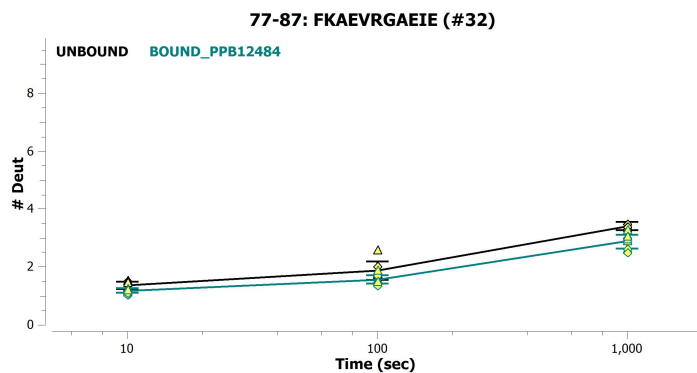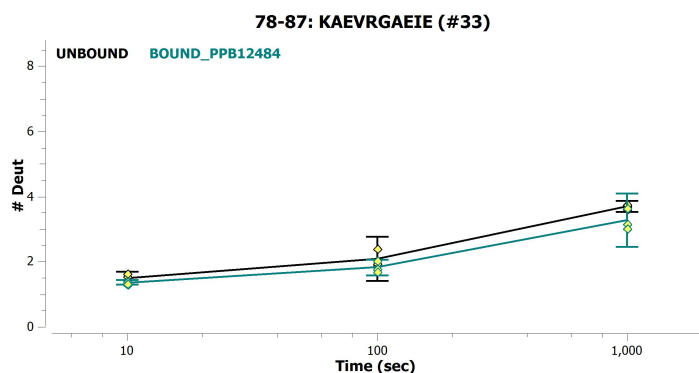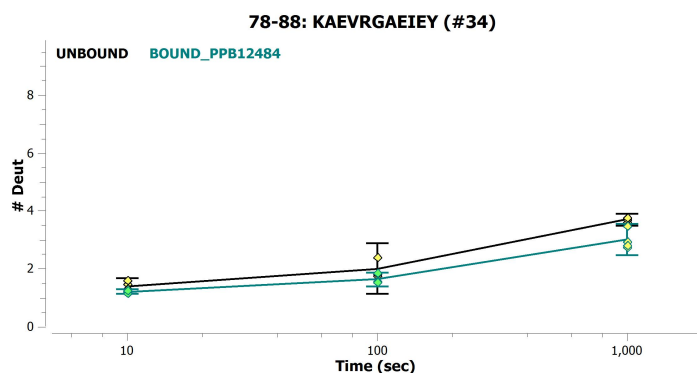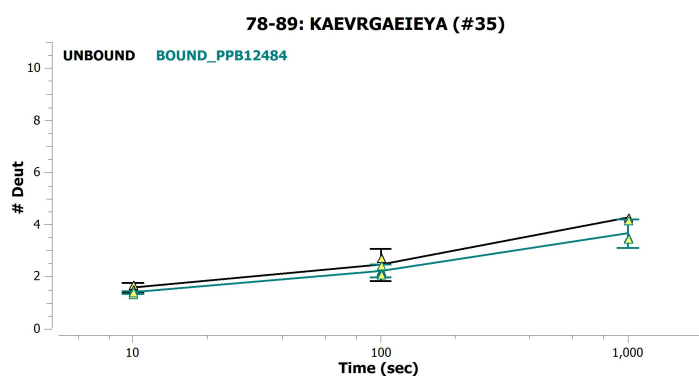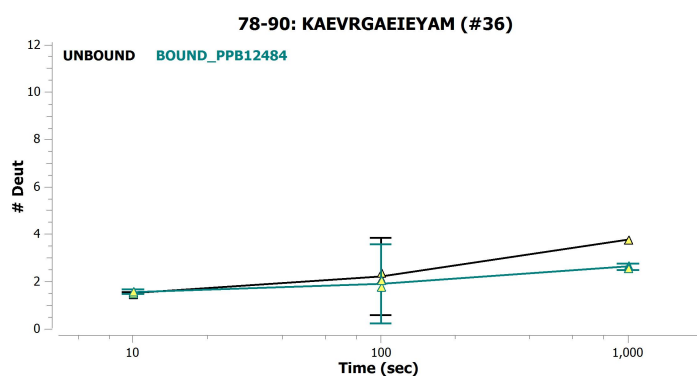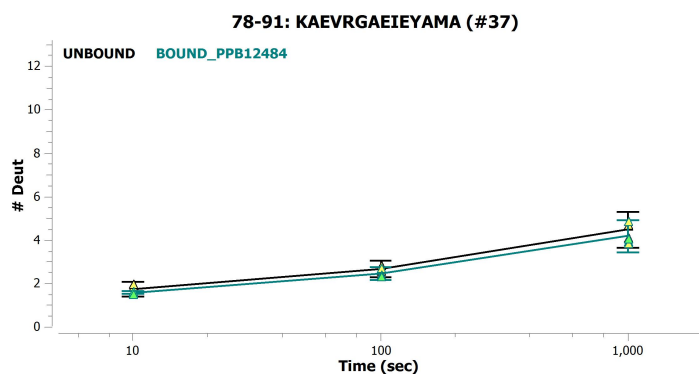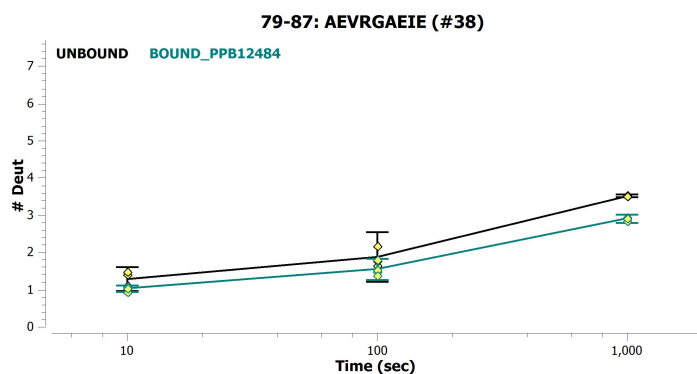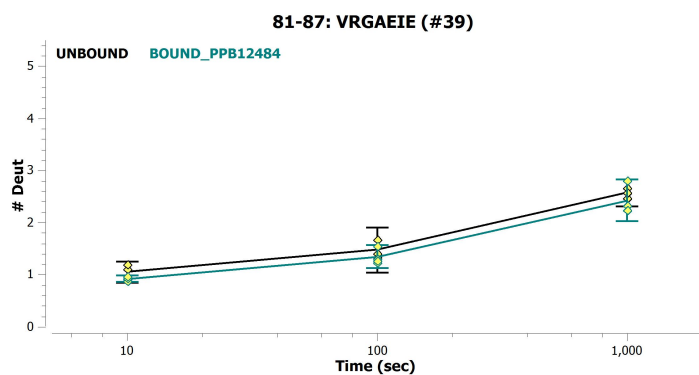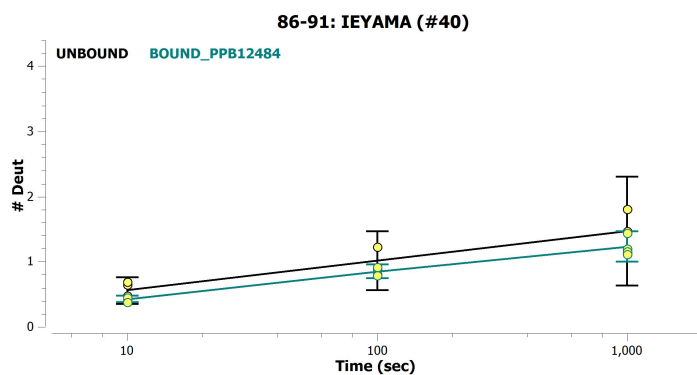

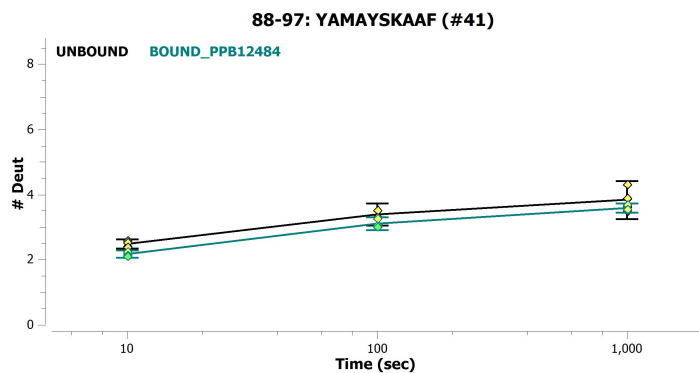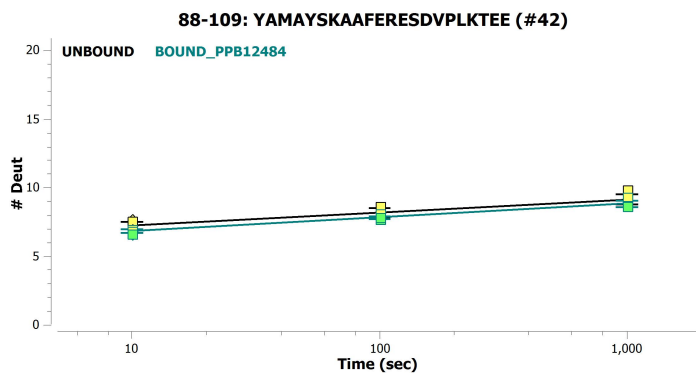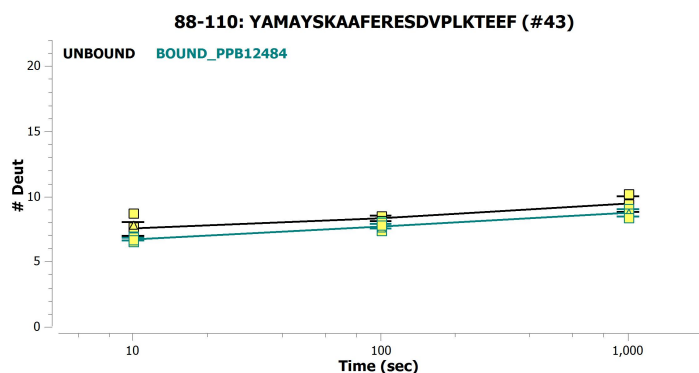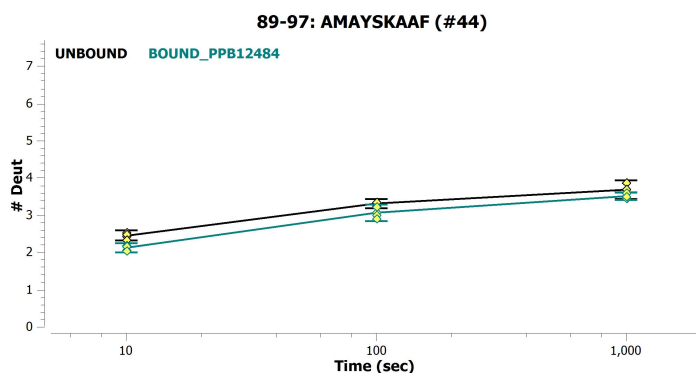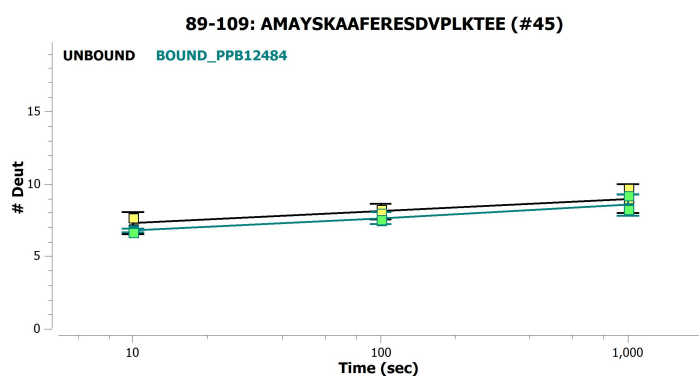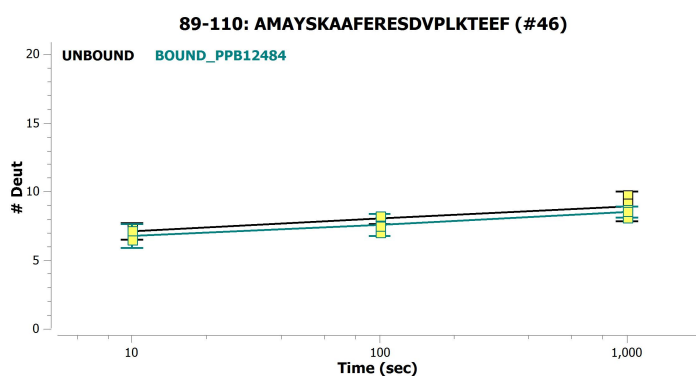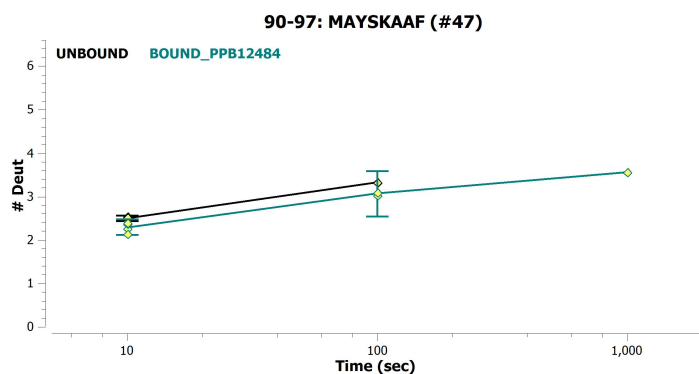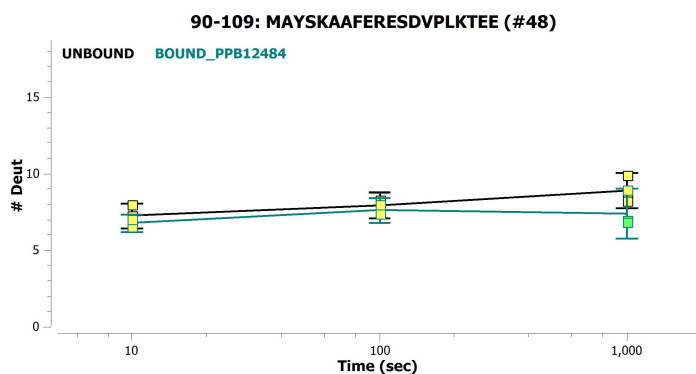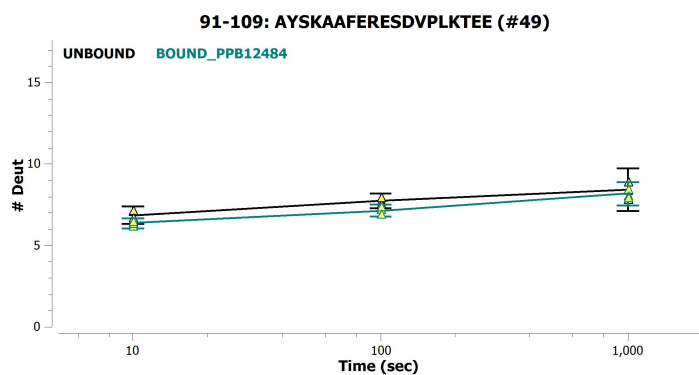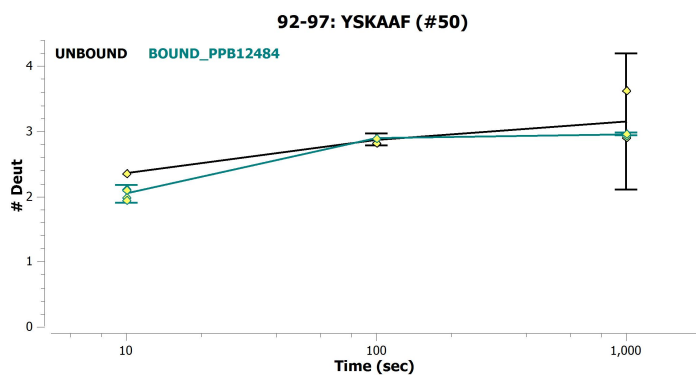

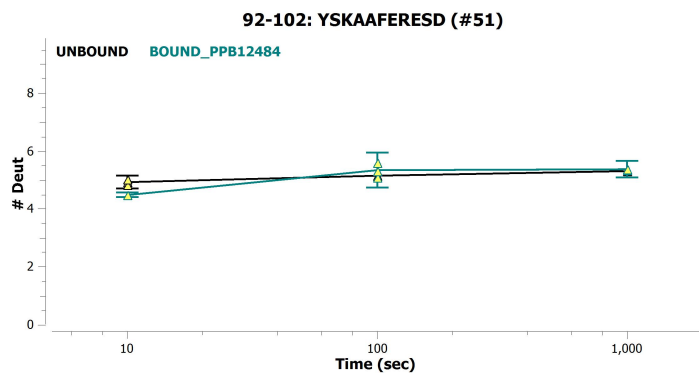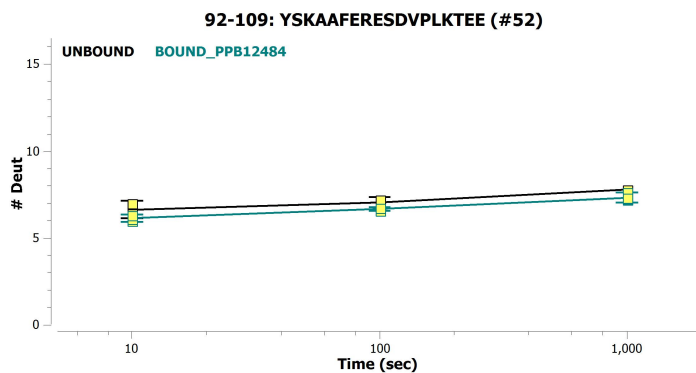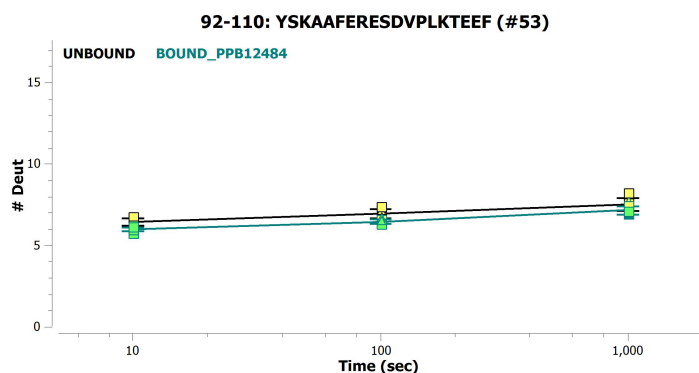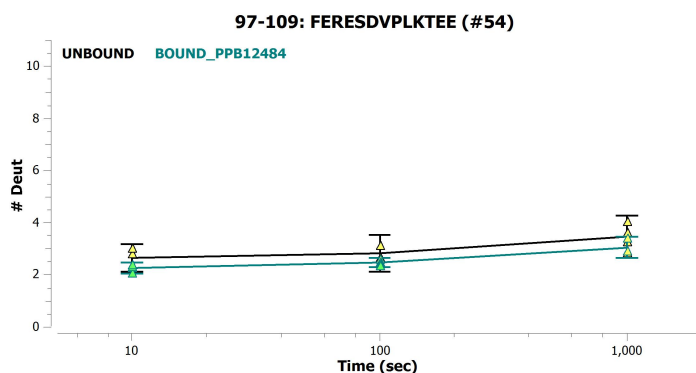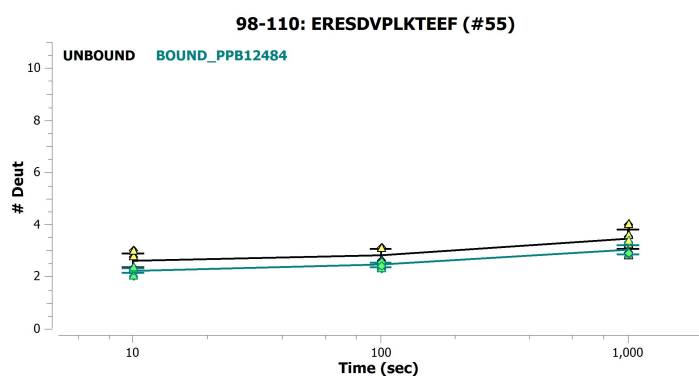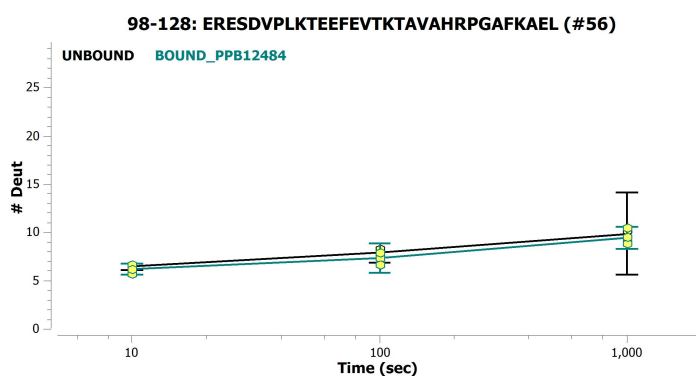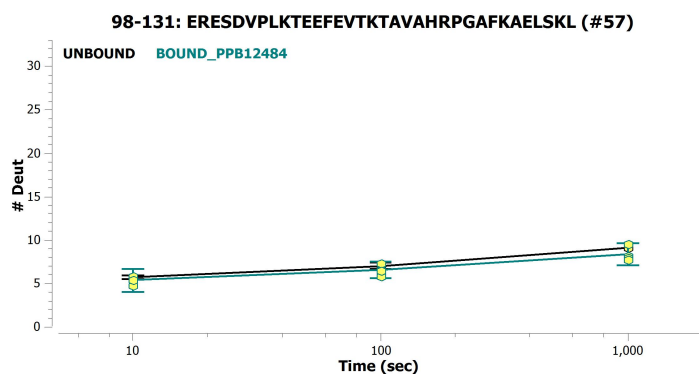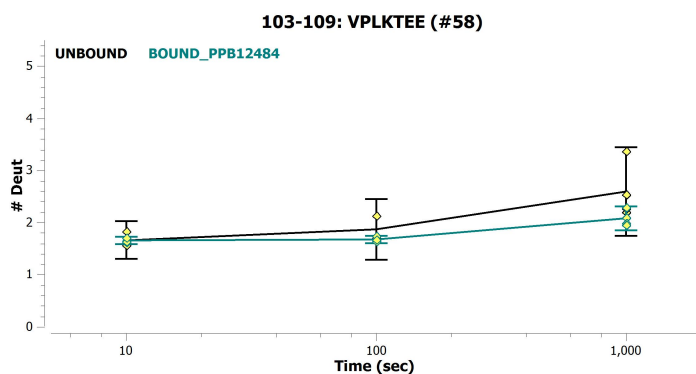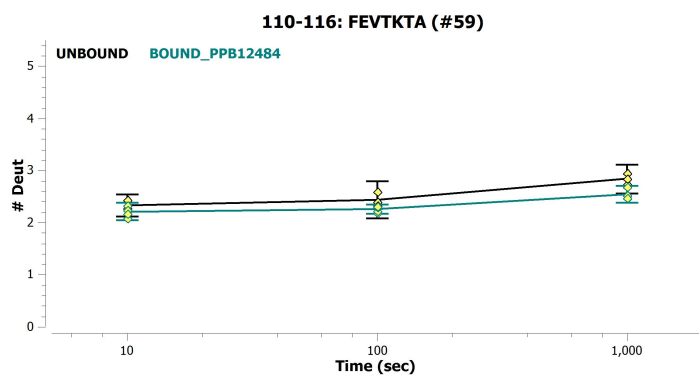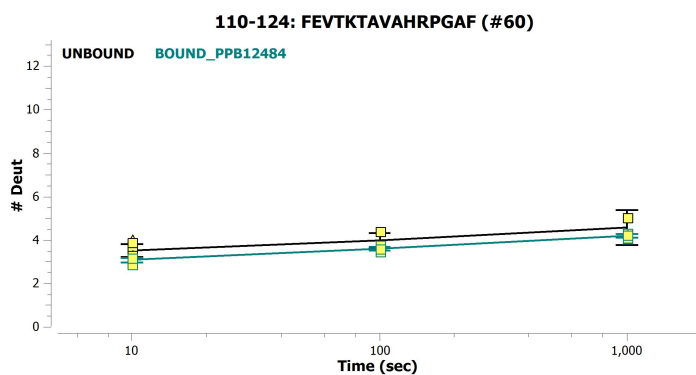

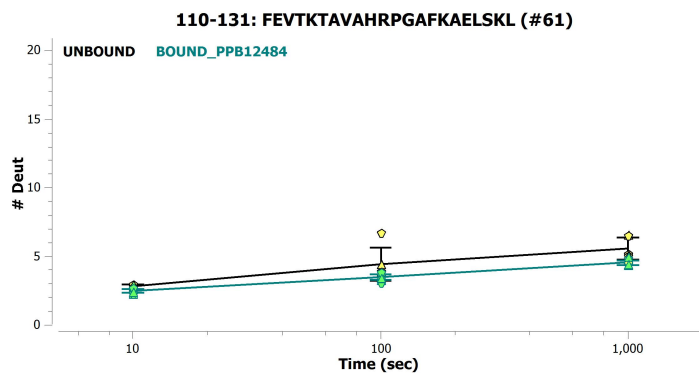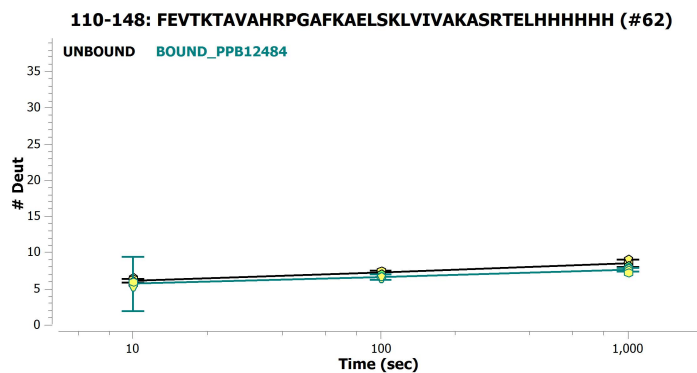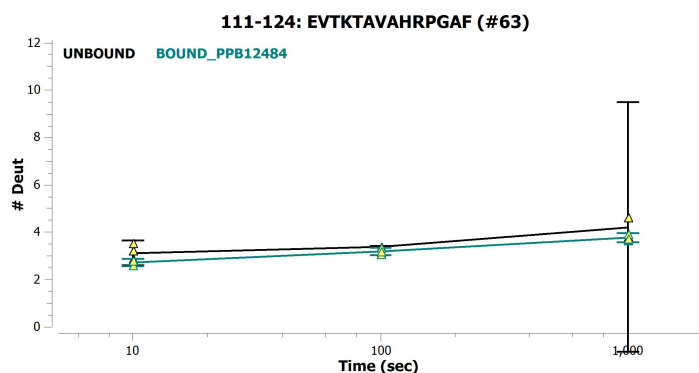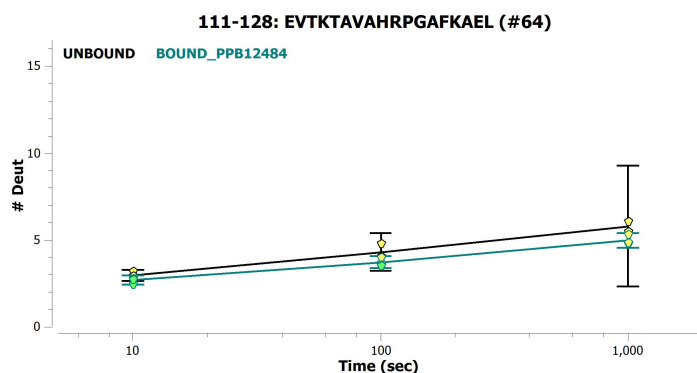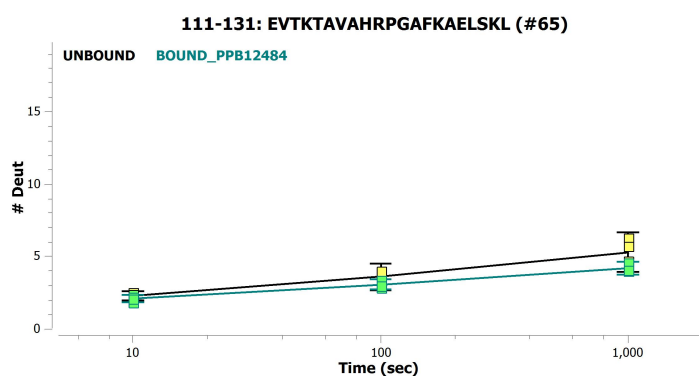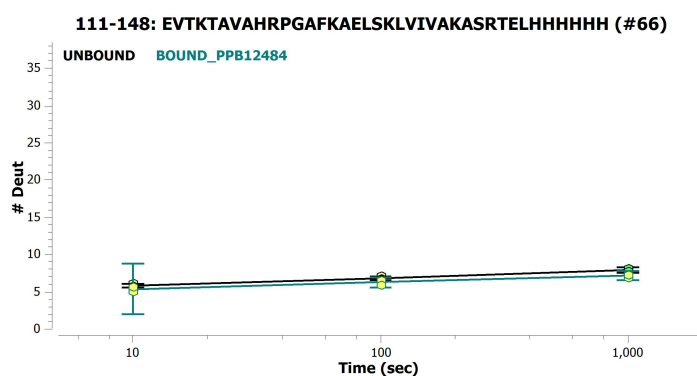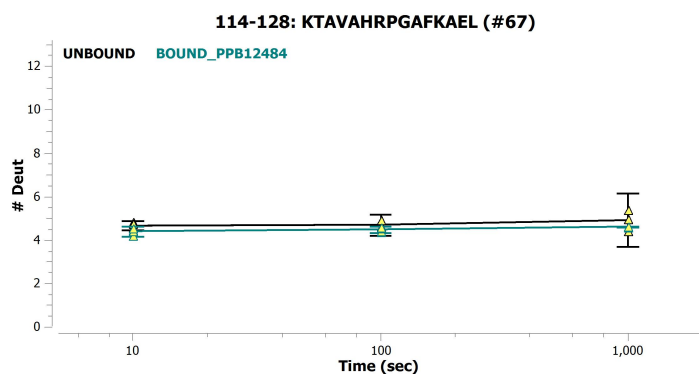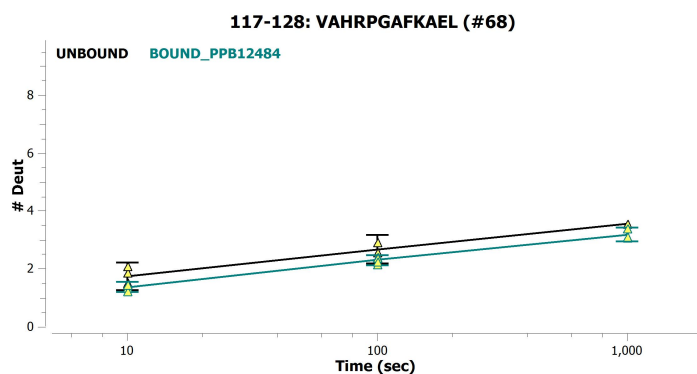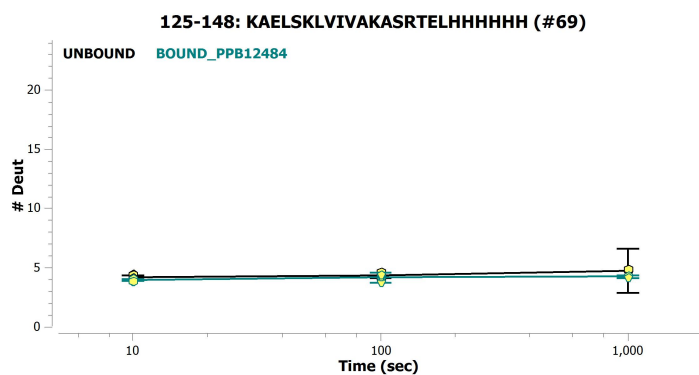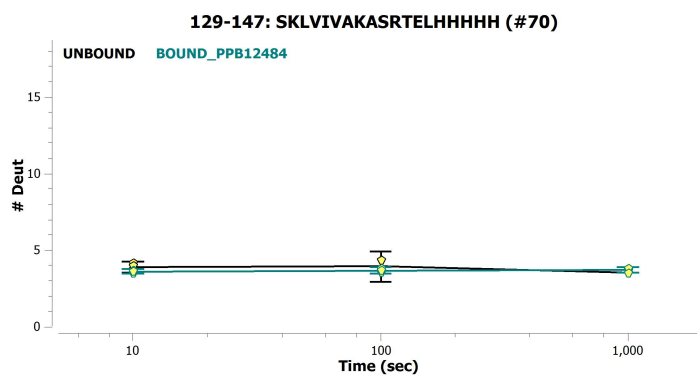

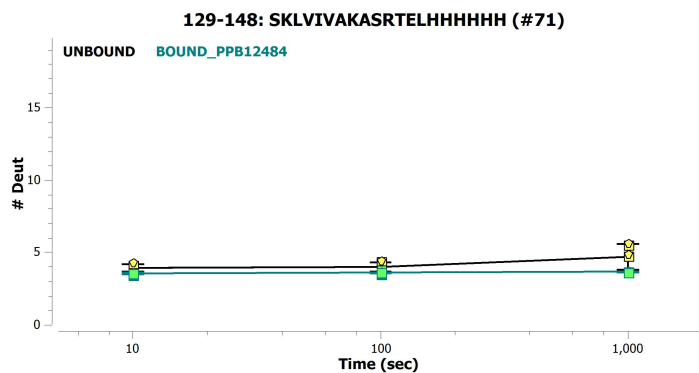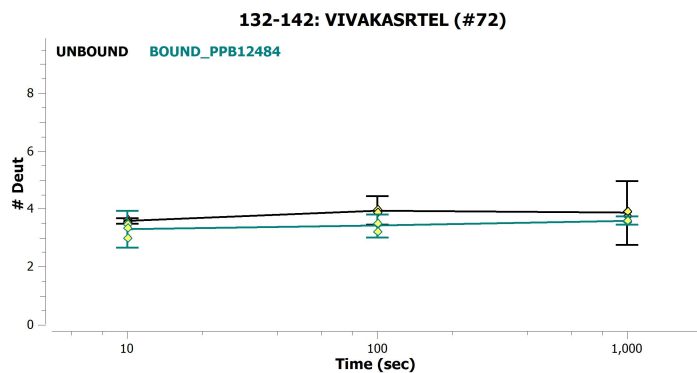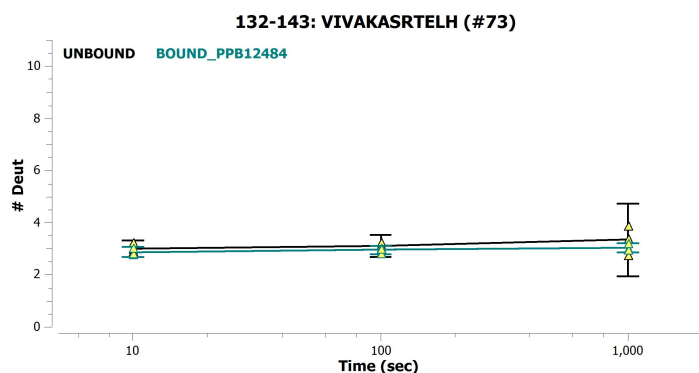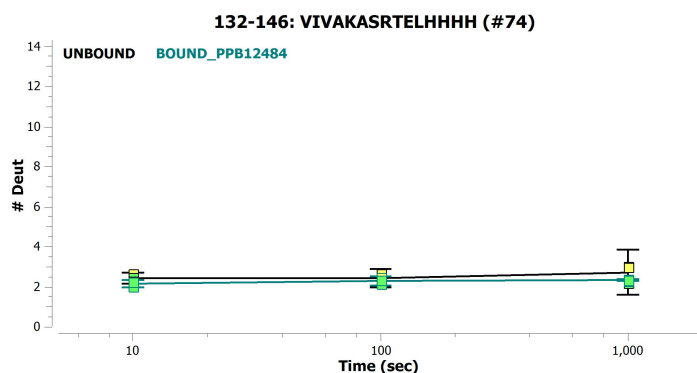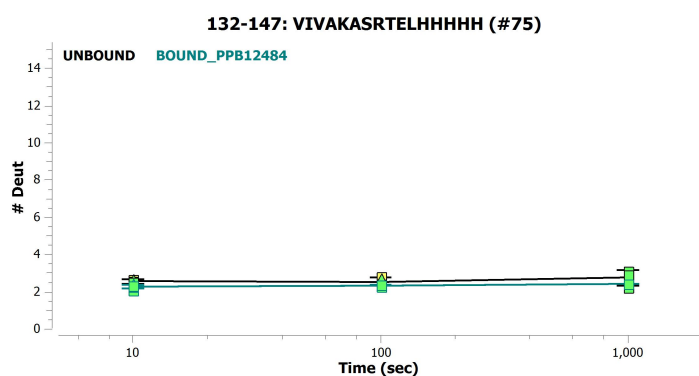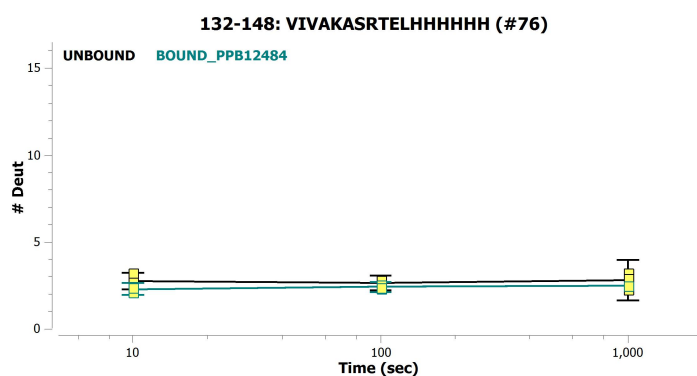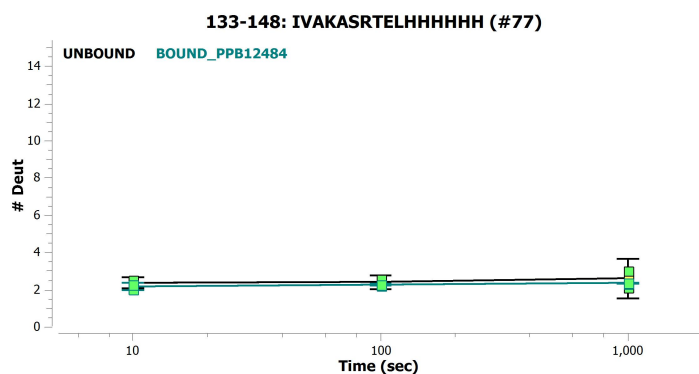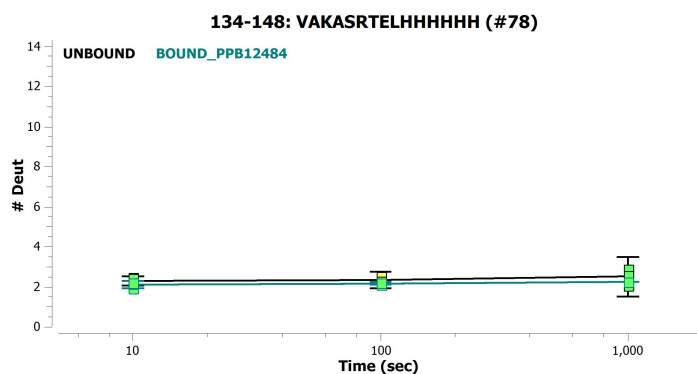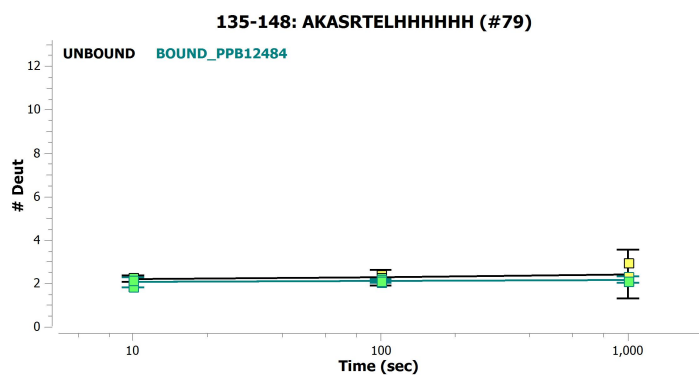

Supplement: Supplementary file 3 — Supplementary Data 1 [file 41467_2019_13343_MOESM3_ESM.pdf]
