# Supplementary material for: Crystal structure and receptor-interacting residues of MYDGF — a protein mediating ischemic tissue repair
Source: Nat Commun. 2019 Nov 26;10:5379. doi: 10.1038/s41467-019-13343-7 (PMC6879528; doi:10.1038/s41467-019-13343-7)
Supplement: Supplementary file 4 — Reporting Summary [file 41467_2019_13343_MOESM4_ESM.pdf]

nature research

Corresponding author(s): Herbert Nar

Last updated by author(s): Aug 27, 2019

Reporting Summary

Nature Research wishes to improve the reproducibility of the work that we publish. This form provides structure for consistency and transparency in reporting. For further information on Nature Research policies, see Authors & Referees and the Editorial Policy Checklist .

Statistics

For all statistical analyses, confirm that the following items are present in the figure legend, table legend, main text, or Methods section.

n/a	Confirmed	
☐	☒	The exact sample size (*n*) for each experimental group/condition, given as a discrete number and unit of measurement
☒	☐	A statement on whether measurements were taken from distinct samples or whether the same sample was measured repeatedly
☐	☒	The statistical test(s) used AND whether they are one- or two-sided<br>*Only common tests should be described solely by name; describe more complex techniques in the Methods section.*
☒	☐	A description of all covariates tested
☐	☒	A description of any assumptions or corrections, such as tests of normality and adjustment for multiple comparisons
☐	☒	A full description of the statistical parameters including central tendency (e.g. means) or other basic estimates (e.g. regression coefficient) AND variation (e.g. standard deviation) or associated estimates of uncertainty (e.g. confidence intervals)
☐	☒	For null hypothesis testing, the test statistic (e.g. *F*, *t*, *r*) with confidence intervals, effect sizes, degrees of freedom and *P* value noted<br>*Give P values as exact values whenever suitable.*
☒	☐	For Bayesian analysis, information on the choice of priors and Markov chain Monte Carlo settings
☒	☐	For hierarchical and complex designs, identification of the appropriate level for tests and full reporting of outcomes
☒	☐	Estimates of effect sizes (e.g. Cohen's *d*, Pearson's *r*), indicating how they were calculated

Our web collection on statistics for biologists contains articles on many of the points above.

Software and code

Policy information about availability of computer code

Data collection	DA+ (PSI)
Data analysis	autoPROC, XDS, STARANISO, Aimless, autoSharp, SHELXD, Coot ,autoBuster, Phaser-MR, ImageJ, GraphPadPrism 6.0

For manuscripts utilizing custom algorithms or software that are central to the research but not yet described in published literature, software must be made available to editors/reviewers. We strongly encourage code deposition in a community repository (e.g. GitHub). See the Nature Research guidelines for submitting code & software for further information.

Data

Policy information about availability of data

All manuscripts must include a data availability statement. This statement should provide the following information, where applicable:

- Accession codes, unique identifiers, or web links for publicly available datasets
- A list of figures that have associated raw data
- A description of any restrictions on data availability

All X-ray structures and related data will be deposited to the PDB upon publication. PDB IDs are provided in the manuscript..

Field-specific reporting

Please select the one below that is the best fit for your research. If you are not sure, read the appropriate sections before making your selection.

☒ Life sciences ☐ Behavioural & social sciences ☐ Ecological, evolutionary & environmental sciences

For a reference copy of the document with all sections, see nature.com/documents/nr-reporting-summary-flat.pdf

Life sciences study design

All studies must disclose on these points even when the disclosure is negative.

Sample size	No statistical techniques were used to determine the sample size. Endothelial cell migration assay and p38 was at least performed in three independent replicates. The reproducibility was good. An identical sample size is typical for such experiments and has been published several times in a similar form.
Data exclusions	No data was excluded from the results.
Replication	All attempts at replication were successful.
Randomization	No research animal, human research participants or clinical studies are involved in our study. Experimental conditions were randomized on multititer plates for the endothelial cell migration and tube formation assays.
Blinding	Endothelial cell migration and tube formation assays were analyzed in a blinded manner.

Reporting for specific materials, systems and methods

We require information from authors about some types of materials, experimental systems and methods used in many studies. Here, indicate whether each material, system or method listed is relevant to your study. If you are not sure if a list item applies to your research, read the appropriate section before selecting a response.

Materials & experimental systems

n/a	Involved in the study
☐	☒ Antibodies
☒	☐ Eukaryotic cell lines
☒	☐ Palaeontology
☒	☐ Animals and other organisms
☒	☐ Human research participants
☒	☐ Clinical data

Methods

n/a	Involved in the study
☒	☐ ChIP-seq
☒	☐ Flow cytometry
☒	☐ MRI-based neuroimaging

Antibodies

Antibodies used	Previously unpublished Antibody8 / Fab8 fragment. The sequence is indicated in the method section.Western Blotting: p38 mitogen-activated protein kinase (MAPK) (clone D13E1, #8690); P-p38 MAPK (T180/Y182) (clone 12F8, #4631).
Validation	Physical binding determined by SPR (data not shown), X-ray structure of Fab8-MYDGF complex.
